# Addressing Inpatient Glycaemic Control with an Inpatient Glucometry Alert System

**DOI:** 10.1155/2015/807310

**Published:** 2015-07-28

**Authors:** J. N. Seheult, A. Pazderska, P. Gaffney, J. Fogarty, M. Sherlock, J. Gibney, G. Boran

**Affiliations:** ^1^Clinical Chemistry Department, Adelaide and Meath Hospital, Tallaght, Dublin 24, Ireland; ^2^Department of Medicine, Endocrinology Division, Adelaide and Meath Hospital, Tallaght, Dublin 24, Ireland

## Abstract

*Background*. Poor inpatient glycaemic control has a prevalence exceeding 30% and results in increased length of stay and higher rates of hospital complications and inpatient mortality. The aim of this study was to improve inpatient glycaemic control by developing an alert system to process point-of-care blood glucose (POC-BG) results. *Methods*. Microsoft Excel Macros were developed for the processing of daily glucometry data downloaded from the Cobas IT database. Alerts were generated according to ward location for any value less than 4 mmol/L (hypoglycaemia) or greater than 15 mmol/L (moderate-severe hyperglycaemia). The Diabetes Team provided a weekday consult service for patients flagged on the daily reports. This system was implemented for a 60-day period. *Results*. There was a statistically significant 20% reduction in the percentage of hyperglycaemic patient-day weighted values >15 mmol/L compared to the preimplementation period without a significant change in the percentage of hypoglycaemic values. The time-to-next-reading after a dysglycaemic POC-BG result was reduced by 14% and the time-to-normalization of a dysglycaemic result was reduced from 10.2 hours to 8.4 hours. *Conclusion*. The alert system reduced the percentage of hyperglycaemic patient-day weighted glucose values and the time-to-normalization of blood glucose.

## 1. Introduction

Hyperglycaemia and hypoglycaemia are prevalent in the inpatient setting. A recent analysis of hospital glucometry data by Bersoux and Cook from January to December 2012 was conducted on 51.4 million measurements from 2.6 million inpatients. The authors found that the prevalence of hypoglycaemia (<4 mmol/L or 70 mg/dL) was 6.1% in non-ICU patients and 5.6% in ICU patients, while the prevalence of hyperglycaemia (>10 mmol/L or 180 mg/dL) was substantially higher at 32% in non-ICU patients and 28% in ICU patients. The mean point-of-care blood glucose (POC-BG) was 167 mg/dL for non-ICU patients and 170 mg/dL for ICU patients. The authors concluded that increased hospital participation in data collection was needed for the development of optimal practices to manage inpatient dysglycaemia [1].

Numerous studies have shown that improvement in glycemic control results in lower rates of hospital complications in general medicine and surgery patients [2–6]. In one study, newly discovered hyperglycaemia was associated with a higher in-hospital mortality rate (16%) compared with those patients with a prior history of diabetes (3%) and subjects with normoglycaemia (1.7%; both *p* < 0.01) [2].

At the opposite end of the glycaemic scale, studies have shown that in-hospital secondary hypoglycaemia increases inpatient mortality, likelihood of readmission, and length of stay [7–9].

Certain clinical situations increase the risk of dysglycaemia during a hospital admission. These include changes in caloric or carbohydrate intake especially “nil by mouth” status or total parenteral nutrition [10]; use of diabetogenic medications like corticosteroids [11]; failure of medical staff to make adjustments to glycemic therapy based on daily blood glucose (BG) patterns [12]; prolonged use of sliding scale insulin regimens [13]; lack of coordination between insulin therapy, blood glucose monitoring, and meals [14]; issues relating to patient transfer from one ward location to another [14]; and medical transcription and dispensing errors [15].

There is evidence that poor inpatient glycaemic control is underrecognized, underreported, and suboptimally managed and that proactive assessment of inpatients' glycaemic status and aggressive treatment approaches are required [16–19]. The American Association of Clinical Endocrinologists/American Diabetes Association (AACE/ADA) recommendations stress five key facets: identification of inpatient dysglycaemia; establishing a multidisciplinary team approach to diabetes management in all hospitals; implementation of structured protocols for aggressive control of BG in both ICUs and other hospital settings; educational programs for all hospital personnel caring for people with diabetes; and planning for a smooth transition to outpatient care with appropriate diabetes management [20, 21].

Current networked point-of-care technology offers a novel way to address the problem of inpatient dysglycaemia and clinical inertia.

Our hospital conducted an audit of inpatient glucometry results from our Accu-Chek Inform II [22] meters and Cobas IT [23] in October 2012 in order to review the identification and management of patients with hypoglycaemia (<4 mmol/L) and with moderate (15–20 mmol/L) and severe (>20 mmol/L) in-hospital hyperglycaemia (see Supplementary Appendix 1 in Supplementary Material available online at http://dx.doi.org/10.1155/2015/807310). 3.4% of readings were in the hypoglycaemic range and 10% of readings were in either moderate or severe hyperglycaemic ranges. On average 12 patients per day had POC-BG results in the moderate-severe hyperglycaemic range, with more than 4 times that number having values >10 mmol/L. The mean time-to-next POC-BG test in a patient with a dysglycaemic value was almost 6 hours with a range of 2 minutes to 33 hours and the mean time-to-normalization of blood glucose after a dysglycaemic result was approximately 10 hours with a range of 1 to 76 hours.

We aimed to develop an alert system where all POC-BG measurements within the moderate-severe hyperglycaemic and hypoglycaemic ranges would be reviewed and notified to the relevant ward teams, nursing staff, and Diabetes Consult service. The metric chosen to analyse glucometry data was the patient-day model, which has been shown to most faithfully reflect the quality of inpatient glycemic control [24]. We aimed to reduce the percentage of patient-day weighted POC-BG levels above 15 mmol/L by at least 20% with a less than 5% increase in levels less than 4 mmol/L. We also aimed to reduce the time-to-next-reading and the time-to-normalization after a dysglycaemic result by 20%.

## 2. Methods

### 2.1. Networked Glucometry System

The glucometers used in this study were the Accu-Chek Inform II system (Roche Diagnostics, Basel, Switzerland) [22]. All hospital glucometers are connected via WLAN to Cobas IT 1000 (Roche Diagnostics, Basel, Switzerland), allowing storage of all information and central management of all meters and data in line with laboratory regulations [23].

### 2.2. Software Development

Microsoft Excel version 2013 (Microsoft, WA, USA) and Visual Basic version 13 (Microsoft, WA, USA) were used to develop Excel Macros for processing of glucometry results from the Adelaide and Meath Hospital, Dublin, Ireland. An explanation of the Visual Basic Code for the Macros is available in the Supplementary Appendix. A sample anonymised data file and a working version of the Macro-Enabled Workbook is also available online (https://app.box.com/s/kk0ka29wmuwk855hdbq4yfqvh7nshfnp).

POC-BG data were classified based on local hospital standards: hypoglycaemia (<4 mmol/L), normoglycaemia (4–10 mmol/L), mild hyperglycaemia (10–15 mmol/L), moderate hyperglycaemia (15–20 mmol/L), and severe hyperglycaemia (>20 mmol/L). Alerts were generated for any hypoglycaemic result and for any result in the moderate-severe hyperglycaemia range.

### 2.3. Study Design

This study was a 60-day prospective study on the impact of a glucometry alert system on hospital dysglycaemia, started on 11 April 2014. There was no Diabetes Registrar rostered to the project for a 14-day period from 05 May 2014 to 18 May 2014; care proceeded as per the preimplementation phase during this period.

#### 2.3.1. Study Approval

Approval was granted by the hospital Diabetes Consult team, hospital management board, laboratory manager, and information technology department. This project did not require submission to the ethics committee since it was aiming to improve the use of routine clinical data.

#### 2.3.2. Integration with Diabetes Consult Team

Our Diabetes Consult service requested that the cutoff for review of a patient be 15 mmol/L rather than the ADA/AACE recommendation of 12 mmol/L since their capacity for reviewing patients was limited to 12–15 patients per day. There are many cutoffs used for classifying a glucose result as hypoglycaemic. Based on audit data showing that only 3.4% of our hospital's results were less than 4 mmol/L, the cutoff for hypoglycaemic alerts was set at this level. The touchscreen tablet was used by the Diabetes Consult Registrar to highlight the hospital patients with poor glycaemic control. The Consult Registrar reviewed the patient's history, hospital stay, and insulin regimen. The alert system was used on weekdays only since there was no Consult service on weekends.

#### 2.3.3. Glucose Alerts on Laboratory Information System

The point-of-care manager, Chemical Pathology trainee, or another member of the biochemistry staff performed daily uploads of dysglycaemic results to WinPath (Clinisys Group, Surrey, UK), our laboratory information system. A Glucose Alert (GA) was added to WinPath for every dysglycaemic result with the following comment:

Please review this patient's in-hospital glycaemic control. We have noted episodes of hypoglycaemia (<4 mmol/L) and/or hyperglycaemia (>15 mmol/L) on the point-of-care glucometry alert system. Refer to the hospital guidelines on glycaemic management and/or request a consult from the Hospital Diabetes Team.

The glucose alert uploads to the LIS were performed only on weekdays during the study period.

### 2.4. Statistical Methods

Data downloaded from the Cobas IT 1000 database were analysed using Stata version 13 (Statacorp, TX, USA). POC-BG results were analysed for the pre- and postimplementation periods, from 10 February 2014 to 10 April 2014 and from 11 April 2014 to 9 June 2014, respectively. Results from the acute care wards, emergency department, and pediatrics and outpatient wards were omitted because these have a substantially different patient demographic and/or are not representative of inpatient glycaemic control.

Summary statistics were obtained for the number of POC-BG results every day and the number and percentage of results <4 mmol/L and >15 mmol/L. The percentage of patient-day weighted mean blood glucoses <4 mmol/L and >15 mmol/L was calculated. Unequal variance one-sided *t*-tests were done to compare mean percentages of hypoglycaemia, hyperglycaemia, hypoglycaemic patient-days, and hyperglycaemic patient-days pre- and postpractice change. The daily mean patient-day weighted POC-BG values were displayed using line graphs with Lowess (locally weighted scatterplot smoothing) lines to show trends over time.

For any POC-BG value <4 mmol/L or >15 mmol/L, the time-to-next-reading and the time-to-normalization of glucose were calculated. If the time between measurements was found to be greater than 72 hours, the patient was assumed to have been either discharged, admitted to ITU, or had serum blood glucoses analysed in the laboratory or on a blood gas analyzer; the time-to-next-reading was taken to be 72 hours in these cases. Comparisons of pre- and postpractice change were made using unequal variance one-sided *t*-tests, histograms, and normal density plots. The proportions of time values greater than 6 hours and 12 hours (time-to-next-reading) or greater than 12 hours and 24 hours (time-to-normalization) were also compared.

Line graphs of the number of POC-BG tests performed per day and the percentage of mean patient-day weighted POC-BG values >15 mmol/L were plotted to investigate trends in glycaemic control and glucometry use over weekends.

### 2.5. Data Security and Confidentiality

The Health Information Technical Standards of the Irish Health Information and Quality Authority (HIQA) were reviewed and followed closely to ensure data security and patient confidentiality [25]. The Head of the Information and Communications Technology Department was also consulted on issues relating to data security.

## 3. Results

In total, 45929 POC-BG results were downloaded from the Cobas IT database for the 120-day analysis period. After removing results from outpatient, paediatric and emergency departments, a total of 13992 results were analyzed for the preimplementation period compared to 14249 in the postimplementation period. There was no statistically significant difference between the numbers of POC-BG values, patient-day values, or patients for the two periods (see Table 1).

In total, there were 2023 dysglycaemic values and 272 patients reviewed by the Diabetes Consult Registrar after implementation. This corresponded to approximately 4 new patients and 10 known patients (from consults or from prior review) per day.

**Figure 1:**
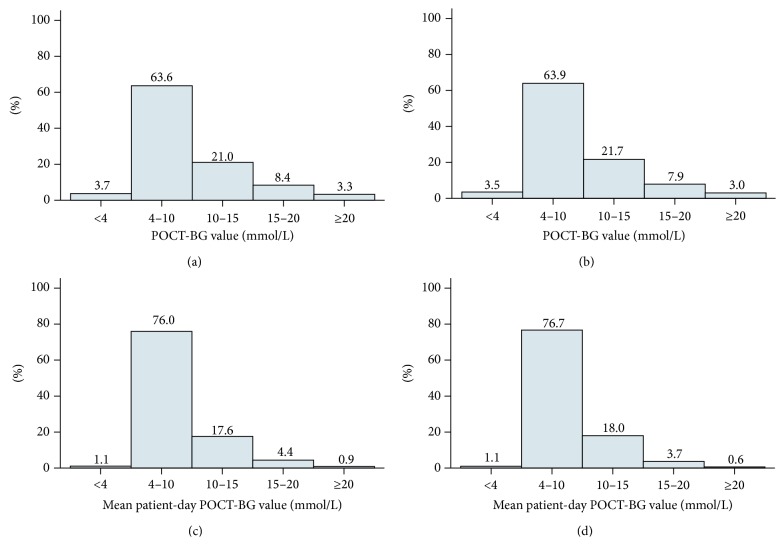
Vertical bar graphs showing the percentage of results by glucose level: (a) preimplementation POC-BG values; (b) postimplementation POC-BG values; (c) preimplementation mean patient-day weighted POC-BG values; and (d) postimplementation mean patient-day weighted POC-BG values.

**Figure 2:**
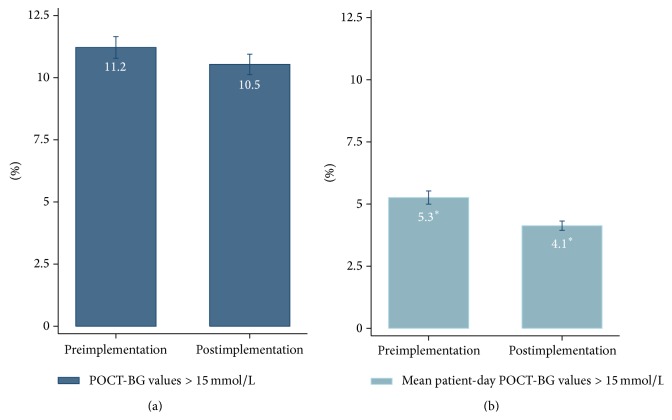
Vertical bar graphs showing the percentage of hyperglycaemic (>15 mmol/L) (a) POC-BG values and (b) mean patient-day weighted POC-BG values. There was a significant reduction (greater than 20%) in mean patient-day weighted POC-BG values > 15 mmol/L after implementation of the alert system. ^*∗*^ *p* < 0.05.

The frequency distributions of POC-BG values and mean patient-day POC-BG values by glycaemic level for the pre- and postimplementation phases are shown in Figure 1. Compared to the preimplementation phase, the percentage of POC-BG values in the moderate-severe hyperglycemia range (>15 mmol/L) decreased by 6.25% (*p* = 0.123). There was a 22.6% reduction in the percentage of patient-day weighted POC-BG values >15 mmol/L (*p* < 0.001), as shown in Figure 2. The percentage of hypoglycaemic values and hypoglycaemic patient-day weighted values did not change significantly. While there was a reduction in the percentage of moderate and severe hyperglycaemic values, there was an increase in the percentage of mild hyperglycaemic values but not to the same degree.

A supplementary analysis was performed after omitting the 14-day period from 05 May to 18 May when no Registrar cover was available to review patients flagged by the alert system. After correcting for this protocol deviation, there was a reduction in POC-BG values >15 mmol/L from 11.2% to 10.4% (*p* = 0.103) and in the percentage of patient-day weighted POC-BG values >15 mmol/L from 5.3% to 4.0% (31% relative reduction, *p* < 0.001) with no change in the percentage of hypoglycaemic values and hypoglycaemic patient-day weighted values (*p* = 0.309 and *p* = 0.176). Furthermore, there was a greater reduction in the mean hospital patient-day weighted POC-BG from 8.22 mmol/L to 8.06 mmol/L (*p* < 0.01).

**Figure 3:**
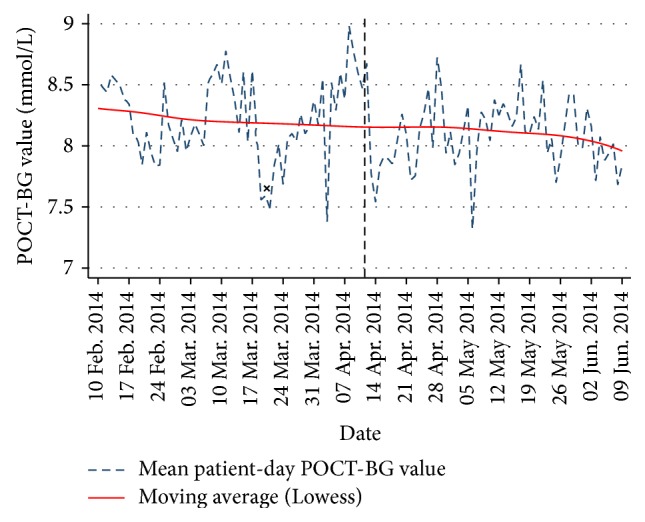
Line graph and Lowess moving average of hospital mean patient-day weighted POC-BG value over time. There was a significant decrease after the date of implementation of the alert system, as highlighted by the dashed line. “x” marks the time period when new hospital guidelines were implemented for the management of inpatient dysglycaemia.

Figure 3 shows the daily change in mean patient-day weighted POC-BG values for the entire study. During the preimplementation phase, the mean hospital value was approximately 8.22 mmol/L. There was a statistically significant reduction to 8.09 mmol/L after the alert system was implemented (*p* < 0.01), as shown in Table 1. The mean patient-day weighted POC-BG value for the hospital plateaued between 05 May and 19 May when the Registrar was not available to review patients. There was a similar trend in mean POC-BG value but this was not statistically significant (not shown here). The “x” in Figure 3 marks the time period when new guidelines were implemented for the management of inpatient dysglycaemia. This coincided with a 1-2-week reduction in the mean patient-day weighted POC-BG value for the hospital; however, the hospital mean reached its previous baseline after this short-lived change.

**Figure 4:**
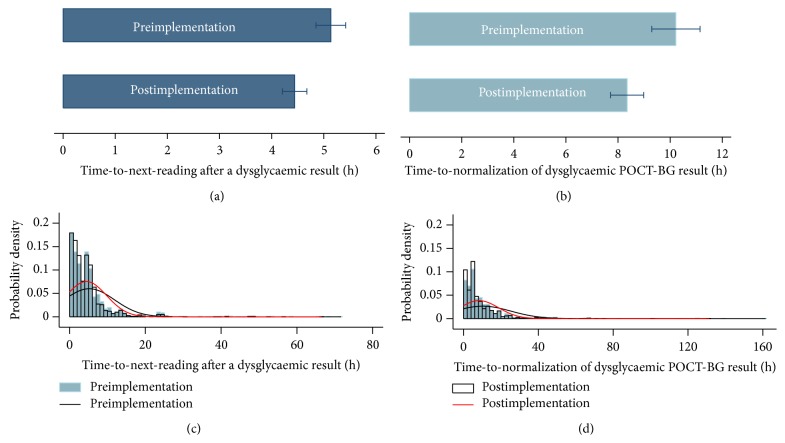
Bar graphs showing mean and 95% CI of (a) time-to-next-reading after dysglycaemic results and (b) time-to-normalization of a dysglycaemic result, before and after implementation. Histograms with probability density plots of (c) time-to-next-reading after a dysglycaemic result and (d) time-to-normalization of a dysglycaemic result, before and after implementation.

There was a significant 14% reduction in the mean time-to-next-reading, from 5.1 hours to 4.4 hours (*p* < 0.01). The density histogram shows a reduction in values over 6 hours, with a shift of the probability density plot to the left (see Figure 4). 25.2% of all times were greater than 6 hours prior to implementation compared to 20.8% after implementation (*p* < 0.001). The mean time-to-normalization of a dysglycaemic POC-BG result decreased by 19% from 10.2 hours to 8.3 hours (*p* < 0.01). The density histogram shows a fall in the number of values greater than 12 hours for this parameter compared to preimplementation. As shown in Table 2, prior to implementation, 25.0% of time-to-normalization values were greater than 12 hours with 7.4% greater than 24 hours compared to 21.5% and 5.1%, respectively, after implementation (*p* < 0.05).

**Figure 5:**
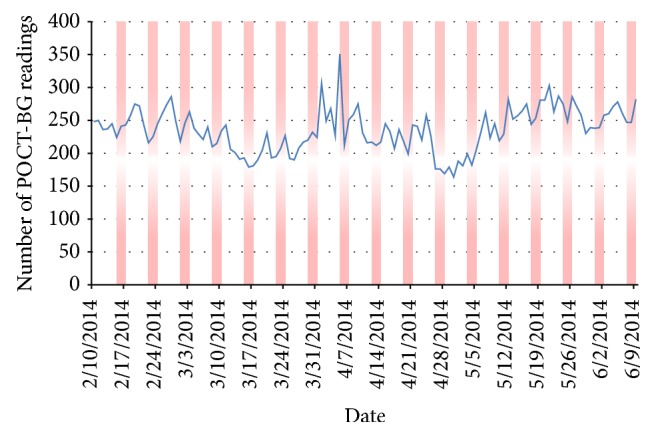
Line graph showing the number of POC-BG tests done by date in all ward locations analysed. Red area bars represent weekends.

The average number of POC-BG tests performed per day was approximately 230. There was a clear trend towards a lower number of tests on weekends with 240 ± 32 tests performed on weekdays compared to 223 ± 33 tests performed on weekends (*p* = 0.008), highlighted by the red bars on Figure 5. Towards the end of the implementation period, there was a rise in the total number of POC-BG tests performed above the previous hospital average.

**Figure 6:**
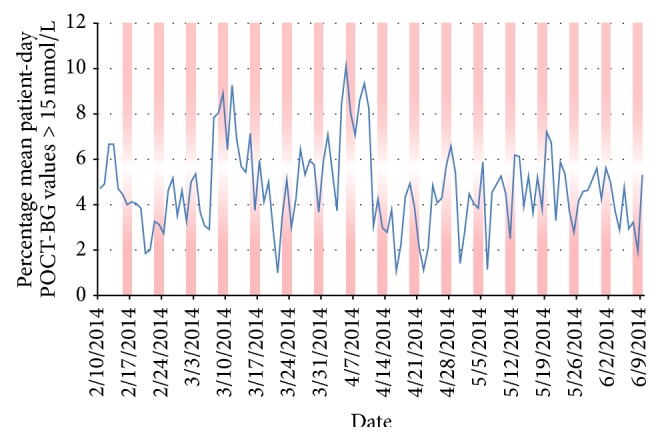
Line graph showing the percentage of mean patient-day weighted POC-BG results greater than 15 mmol/L by date in all ward locations analysed. Red area bars represent weekends.

A trend towards increased proportions of dysglycaemic results on weekends was also noted (indicated by the red bars on Figure 6); this trend was exaggerated in the postimplementation phase. However, there was no statistically significant difference in the proportion of hyperglycaemic results on weekends compared to weekdays. There was also a significant reduction in the coefficient of variation of the percentage of hyperglycaemic results from 40% to 35%; this is visually evident by the stabilization of postimplementation values.

## 4. Discussion

A comprehensive alert system was devised using Visual Basic code and Microsoft Excel Macros to process data and provide charts and tables representing daily glycaemic control for inpatient wards in the hospital. This data was used to provide alerts to hospital teams via the Laboratory Information System and to alert the Diabetes Consult service about patients requiring urgent review.

Data from the preimplementation period agreed with results from our audit in 2012, showing that the percentage of hypoglycaemic results was between 3 and 4%, the percentage of hyperglycaemic results in the mild range was ~20%, and the percentage of results in the moderate-severe hyperglycaemic was approximately 12%. This shows that our rates of dysglycaemia were relatively stable over the previous year.

The implementation of the glucometric alert system achieved our target of a 20% reduction in the percentage of patient-day weighted POC-BG values above 15 mmol/L, the main metric used in this study. Importantly, the percentage of hypoglycaemic results did not change significantly. One of the known complications of strict glycaemic control is an increase in secondary hypoglycaemic events. While addressing the problem of hyperglycaemia, a related goal was to ensure that the proportion of hypoglycaemic results either reduced or remained unchanged and this was achieved by the alert system.

Hypoglycaemic events tend to be managed more proactively in hospital compared to hyperglycaemia for a couple of reasons. Firstly, patients are usually symptomatic when blood glucose levels fall below 3 mmol/L unless they have had frequent hypoglycaemic events in the past or are on beta-blocker therapy, rendering them “hypoglycaemia unaware.” Secondly, the management of hypoglycaemia is much simpler in the initial stages with oral or intravenous glucose formulations or intramuscular glucagon, with an almost immediate rise in blood glucose levels. Hospital and nursing staff have a tendency to be more permissive of mild-moderate hyperglycaemia, employing a watch and wait approach allowing time for the patient's own insulin or hypoglycaemic regimen to work and waiting for consecutive results to be above some arbitrary threshold before alerting hospital teams. This is evident by the much higher rates of hyperglycaemia compared to hypoglycaemia.

The most important benefit of the alert system seems to be the improvement in the time-to-normalization of a dysglycaemic result. This coincided with more frequent POC-BG testing if a result was dysglycaemic, manifested by an increased number of POC-BG tests performed after implementation and a reduction in the time-to-next-reading. The 19% reduction in time-to-normalization and the 14% reduction in mean time-to-next-reading fell short of our 20% target; however, there was a 31% relative reduction in time-to-normalization values greater than 24 hours and a 30% relative reduction in time-to-next-reading values greater than 12 hours. This means that the alert system successfully reduced significant outliers seen in the preimplementation phase. Likely explanations are that the Diabetes Consult service reviewed patients in a more timely fashion and also that more physiological insulin regimens were utilized to normalize dysglycaemic results.

During our 2012 audit, we noticed that the weekend glycaemic control was worse than that seen on weekdays, with higher levels of moderate-severe hyperglycaemia. This problem was inadequately addressed in this study because of a lack of staff coverage on the Diabetes Consult service on weekends. Our results show a trend towards lower numbers of POC-BG tests on weekends; the number of tests performed increased towards the end of the implementation period for unknown reasons. There were also frequent spikes in the percentage of patient-day weighted hyperglycaemic results on weekends and this trend was exaggerated during the implementation phase, likely due to the fact that weekday glycaemic control was improved significantly. The trends observed were not statistically significant; however, they do mirror the trends seen during our previous audit and warrant further investigation and action plans.

Figure 6 shows that the variability in the percentage of hyperglycaemic values was reduced. Before implementation, two peaks in hyperglycaemia were noted; these peaks may be due to the patient population in the hospital at the time. On 21 March 2014, new guidelines for management of dysglycaemia were launched in the hospital and a presentation was given to general medical and surgical teams outlining these guidelines. It is likely that glycaemic control improved in the week following the launch of these new guidelines and that the baseline rate of dysglycaemia in the preimplementation period would have been higher than what was witnessed.

The major limitations of this study related to personnel, financial, and technical issues. The Diabetes Consult service was run by a team of one Consultant and one Registrar and the number of patients that could be reviewed on a daily basis was limited to 10–15. Also, a Consult service based on flagged dysglycaemic results was not provided on weekends and public holidays. It is likely that the percentage of dysglycaemic patient-days would have been significantly lower with more staffing cover. Funding for specialized software development was not available and all technical expertise was local and limited in scope. Uploads to the Laboratory Information System and to the tablets were done twice daily, meaning that there was a significant lag between the time when the glucometry test was performed and the time when the result was uploaded or reviewed by the Diabetes Consult Registrar. Further integration with the current IT framework would allow for instantaneous uploads to the LIS and updates to the interactive reports and charts on the tablets in a push notification fashion.

While this study showed an improvement in glycaemic control, no clinical correlations could be made. Determining changes in length of stay, inpatient mortality, and cost of care by using the alert system was outside the scope of the study and would require significant input by hospital administration. The number of patients in hospital with either a primary or secondary diagnosis of diabetes would be a key metric to monitor over time and control for. Estimates of dysglycaemia could then be corrected for the number of diabetics in each ward location. Additionally, the alert system was only implemented for a 60-day period in this pilot study. Due to fluctuations in the patient profile admitted to hospital, it is possible that the improvements in glycaemic control are artefactual.

Potential areas for improvement would be in the user interface and alert software as well as in the current networking infrastructure. Direct access to the Cobas IT webserver would allow more frequent automatic downloads of data to a network shared folder, which can be accessed on demand by the tablets or any computer in the hospital. Measurement of ketones using point-of-care devices has become more important since the Joint British Diabetes Society (JBDS) issued guidelines in March 2010 recommending ketone (beta-hydroxybutyrate) measurement in the management of DKA [26]. Ketone results from point-of-care devices should also form part of a comprehensive alert system geared especially towards type 1 diabetics.

To determine whether there is a reduction in hospital cost, length of stay, and improved outcomes associated with improved glycaemic control due to the alert system, a six-month to one-year study is needed, with comparison to the previous year. Key metrics like length of stay and inpatient mortality, as well as perioperative outcomes for patients with a primary or secondary diagnosis of diabetes, will need to be recorded rigorously. The longer timeframe of the study will negate the effect of artefactual fluctuations in glycaemic control that are caused by the number of diabetics in hospital and other factors.

## 5. Summary and Conclusions

An automated alert system that allowed the use of site-specific level and time-based criteria for the identification of dysglycaemic results and generation of alerts was successfully developed and deployed in our medium-large sized tertiary care facility. There was a significant improvement in our hospital's glycaemic control, with a reduction in the proportion of hyperglycaemic patient-days and no rebound increase in secondary hypoglycaemia. The alert system also improved the frequency with which glucometry tests were performed in patients with recognized out-of-control glucose results and improved the time-to-normalization of dysglycaemic results. The percentage of patient-day weighted results >15 mmol/L was reduced by greater than 20% and while the reduction in the time-to-normalization of dysglycaemic POC-BG results fell short of our 20% target, we successfully reduced the proportion of values greater than 12 hours by more than 20%. Certain trends in glucometry testing and glycaemic control were also observed, with lower numbers of tests performed and spikes in hyperglycaemia on weekends. Longer-term studies will enable us to determine whether improvements in glycaemic control correlate with improvements in in-hospital morbidity and mortality and reductions in length of stay and hospital costs.

## Supplementary Material

Supplementary Appendix 1 describes the methodology and results of a 5-day audit of inpatient glucometry results from our hospital's Accu-Chek Inform II glucose meters in October, 2012 to determine the rate of hypoglycaemia and hyperglycaemia in our hospital.
Supplementary Appendix 2 provides a detailed description of the Excel Macros that were developed with sample illustrations of how data is processed using the Macros.

## Figures and Tables

**Table 1:** Summary statistics for pre- and postimplementation periods.

Period	Preimplementation	Postimplementation	*p* (*α* = 0.05)
Date range (number of days)	10-02-2014 to 10-04-2014 (60)	11-04-2014 to 09-06-2014 (60)	—
Mean POC-BG value (mmol/L)	9.13 ± 4.57 (1.00–33.30)	9.09 ± 4.47 (0.80–33.30)	0.315
Mean patient-day POC-BG value (mmol/L)	8.22 ± 3.40 (1.80–28.40)	8.09 ± 3.20 (2.80–33.30)	0.010
Number of POC-BG readings	13992	14249	0.237
Number of patient-days with POC-BG readings	5984	5741	0.316
Number of patients with POC-BG readings	816	752	0.186

**Table 2:** Proportion of values for time-to-next-reading and time-to-normalization values above specified thresholds.

Time-to-next-reading after dysglycaemic result			
	Preimplementation	Postimplementation	*p* (1-tailed)
>6 hours	25.20%	20.80%	0.0007
>12 hours	8.60%	6.00%	0.0004

Time-to-normalization of dysglycaemic result			
	Preimplementation	Postimplementation	*p*

>12 hours	25.00%	21.50%	0.032
>24 hours	7.40%	5.10%	0.016
