# Supplementary material for: Addressing Inpatient Glycaemic Control with an Inpatient Glucometry Alert System
Source: Int J Endocrinol. 2015 Jul 28;2015:807310. doi: 10.1155/2015/807310 (PMC4531187; doi:10.1155/2015/807310)
Supplement: Supplementary file 1 — Supplementary Appendix 1 describes the methodology and results of a 5-day audit of inpatient glucometry results from our hospital's Accu-Chek Inform II glucose meters in October, 2012 to determine the rate of hypoglycaemia and hyperglycaemia in our hospital. Supplementary Appendix 2 provides a detailed description of the Excel Macros that were developed with sample illustrations of how data is processed using the Macros. [file 807310.f1.pdf]

SUPPLEMENTARY APPENDIX 1

Clinical (Practice) Audit Report 2012

Your Name Jansen Seheult
Date Audit Commenced 05/10/2012
Date Audit Concluded 09/10/2012
Title Point of Care Testing as a tool for early recognition and management of moderate and severe inpatient hyperglycaemia.

Background

Diabetes Mellitus (DM) is characterized by an elevated blood glucose level. This can be due to insulin resistance (type 2 diabetes mellitus), to type 1 diabetes, or occasionally to secondary causes. Type 2 diabetes accounts for 85-90% of diabetic cases. Worldwide ~ 200 million are known to have Diabetes. Type 2 DM affects about 5% of Irish adults.

This table shows diabetic diagnostic criteria reported by WHO/IDF:

Condition	Fasting	2 hour glucose	HbA_{1c}
	mmol/l(mg/dl)	mmol/l(mg/dl)	%
Normal	<6.1 (<110)	<7.8 (<140)	<6.0
Diabetes mellitus	≥7.0 (≥126)	≥11.1 (≥200)	≥6.5

Apart from the various micro-vascular and macro-vascular complications, there are three life-threatening emergencies in Diabetes Mellitus:

- Diabetic Ketoacidosis [Hyperglycaemia>11.1 mmol/L, pH<7.3, HCO3<15 mmol/L, ketonuria]
- Hyperosmolar Non-Ketotic hyperglycaemia [Hyperglycaemia>30 mmol/L, Serum osmolality>350mmol/kg, no acidosis, no ketonuria.
- Hypoglycaemia [cutoff points vary]

Hypoglycaemia tends to be adequately managed in hospitalized patients due to early autonomic and neuroglycopaenic signs and symptoms, except in patients on concurrent beta-blocker therapy.

Hyperglycaemia is prevalent in the inpatient setting and is associated with increased morbidity and mortality in patients with and without diabetes. Too often hyperglycaemia is underrecognized, underreported, and suboptimally managed. Proactive assessment of inpatients' glycemic status and aggressive treatment approaches are needed. There is a recognized need for improved hyperglycaemia management in inpatients. While Diabetic Care Bundles are available, they are not widely followed and there are numerous problems in managing inpatient hyperglycaemia including:

- Limited access to guidelines or care pathways
- Non-physiologic sliding-scale insulin protocols that result in poor glycemic control or wide fluctuations in glucose levels
- Errors in glucose administration
- Failure to involve Diabetic team in a timely fashion due to lack of integration of services
- Reluctance to implement tight glycaemic control regimens in the inpatient setting due to higher tolerance on the part of medical staff.
- Reluctance to use intravenous or continuous subcutaneous insulin sliding scales.

The issue of glycaemic targets, appropriate education and access to resources, the need for noninvasive blood glucose monitoring, and the role of nursing staff are vital in addressing the above issue.

In this regard, a computerized Point of Care Glucometry System with daily audits of cases of hypo- and hyper-glycaemia, which is integrated with hospital teams, and the Diabetic Service may be a viable solution to targeting the issue of poor inpatient glucose control.

Aim(s) of the audit

The aim of the audit was to review the identification and management of patients with hypoglycaemia and with moderate and severe in-hospital hyperglycaemia.

- Hypoglycaemia [glucose <4.0]
- Moderate hyperglycaemia [glucose 15-20 mmol/L]
- Severe hyperglycaemia [glucose >20 mmol/L]

Objectives

1. To identify all cases of moderate and severe hyperglycaemia
2. To identify all cases of hypoglycaemia
3. To determine whether patient location was related to incidence of hyperglycaemia
4. To determine whether patient location was related to incidence of hypoglycaemia
5. To analyze the time to next glucose reading following the discovery of moderate or severe hyperglycaemia
6. To analyze the time to normalization of glucose following the discovery of moderate or severe hyperglycaemia.

Standards

Management of Hyperglycaemia in Hospitalized Patients in Non-Critical Care Setting: An Endocrine Society Clinical Practice Guideline.

1. Bedside capillary POC testing as the preferred method for guiding ongoing
2. Glycemic management of individual patients.patients without a history of diabetes with BG greater than 7.8 mmol/liter (140 mg/dl) be monitored with bedside point of care (POC) testing for at least 24 to 48 h. Those with BG greater than 7.8 mmol/liter require ongoing POC testing with appropriate therapeutic intervention.
3. For avoidance of hypoglycaemia, we suggest that antidiabetic therapy be reassessed when BG values fall below 5.6 mmol/liter (100 mg/dl). Modification of glucose-lowering treatment is usually necessary when BG

values are below 3.9 mmol/liter (70 mg/dl).

4. POC testing can be discontinued in nondiabetic patients if all BG results are below 7.8 mmol/liter (140 mg/dl) without insulin therapy for a period of at least 24–48 h.
5. Hospitals provide administrative support for an interdisciplinary steering committee targeting a systems approach to improve care of inpatients with hyperglycaemia and diabetes.
6. Each institution establish a uniform method of collecting and evaluating POC testing data and insulin use information as a way of monitoring the safety and efficacy of the glycemic control program.

In May 2009, American Association of Clinical Endocrinologists (AACE) and the American Diabetes Association (ADA) revised their inpatient glycemic targets to 140–180 mg/dL [7.8 -10.0 mmol/L] in the ICU and non-ICU preprandial glucose levels below 140 mg/dL [7.8 mmol/L] and all random glucose levels below 180 mg/dL [10/0 mmol/L].

Methodology

1001 glucose results which had been measured by nurses on ward-based glucometers over a five day period between 05/10/2012 at 00:00 and 09/10/2012 at 12:00 were included in the audit. These were collected from the Cobas IT® Glucometry database (supplied by Roche Diagnostics Limited). In Tallaght Hospital, all ward-based glucometers are interfaced to this database for the purposes of the audit. Only patients with hyperglycaemia > 10.0 mmol/l [AACE/ADA recommendations] were used because the database does not record whether the patients were fasting or post-prandial at the time of the glucose measurements.

The extracted glucose data was analyzed in Microsoft® Excel® Test results were omitted. Data were analyzed by individual glucose readings and then by patient, after omission of duplicate readings each day. Sub-analyses were done by ward location. Cases of moderate and severe hyperglycaemia were also charted temporaneously and summary statistics calculated.

Results

A total of 1001 glucose readings were retrieved from a 5-day period. There were 94 Test readings which were omitted, leaving 907 valid glucose recordings. There were approximately 200 glucometry samples taken each day. The majority of readings [60-87%] fell within the normoglycaemic range. Overall, 3.4% of readings were in the hypoglycaemia range. There were 258 recordings >10.0 mmol/L [i.e. the ADA cutoff for inpatient hyperglycaemia]. Approximately 10% of readings reflected either moderate or severe hyperglycaemia. Overall, only 2.4% of readings were in the severe hyperglycaemia category. The lowest glucose reading was 2.5 mmol/L and there were 10 readings <3.0 mmol/L. Two readings feel within the DKA criteria range [>30 mmol/L], with the highest glucose result being 33.2 mmol/L.

Table S1 showing glucose recordings stratified by glucose level and date of recording.

Glucose Level [mmol/L]	Total	09/10/2012	08/10/2012	07/10/2012	06/10/2012	05/10/2012
	31	0	5	7	6	13
<4.0	(3.4%)	(0.0%)	(2.1%)	(3.3%)	(3.2%)	(6.7%)
	618	66	171	140	125	116
4.0-10.0	(68.2%)	(86.8%)	(70.6%)	(66.4%)	(67.6%)	(60.1%)
	170	7	41	37	36	49
10.0-15.0	(18.7%)	(9.3%)	(16.9%)	(17.5%)	(19.5%)	(25.4%)
	66	3	19	20	13	11
15.0-20.0	(7.3%)	(3.9%)	(7.9%)	(9.5%)	(7.0%)	(5.7%)
	22	0	6	7	5	4
>20.0	(2.4%)	(0.0%)	(2.5%)	(3.3%)	(2.7%)	(2.1%)
	**907**	**76**	**242**	**211**	**185**	**193**

Figure S1 showing glucose recordings for the five day period.

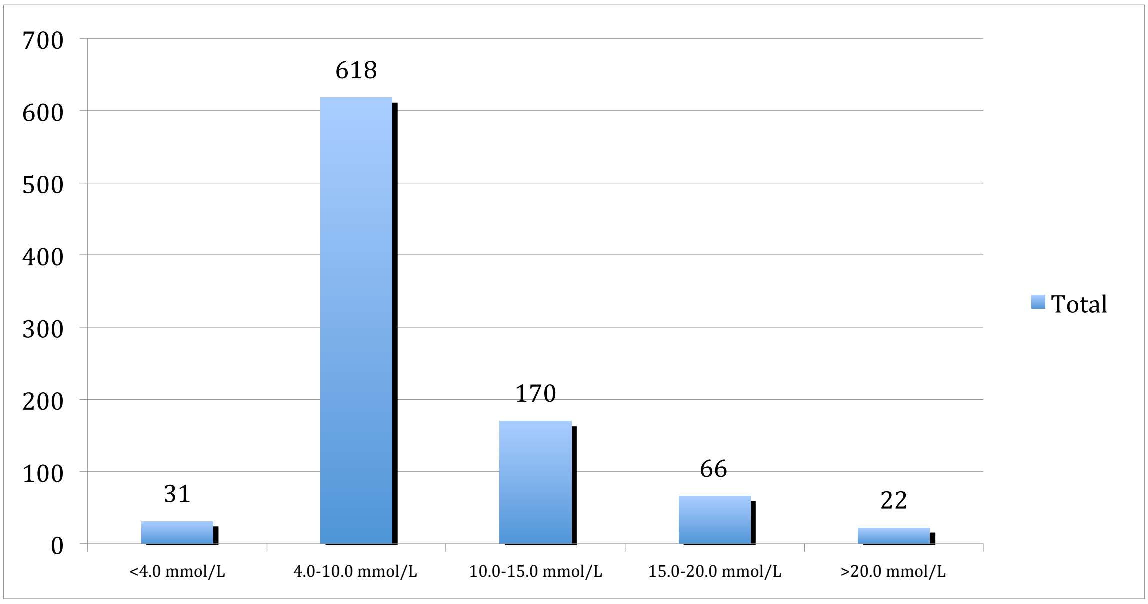

Figures S2 and S3 show glucose recordings stratified by glucose level and by date. They show similar trends, i.e. the majority of readings were in the normoglycaemia range with more patients in the moderate hyperglycaemia range than in the severe hyperglycaemia range. The most recordings were taken on Monday 8th October [26.7%] and Sunday 7th October [23.2%]. The most cases of moderate and severe hyperglycaemia occurred on Sunday 7th October, 20 readings and 7 readings respectively.

Figure S2 showing glucose recordings stratified by glucose level and date.

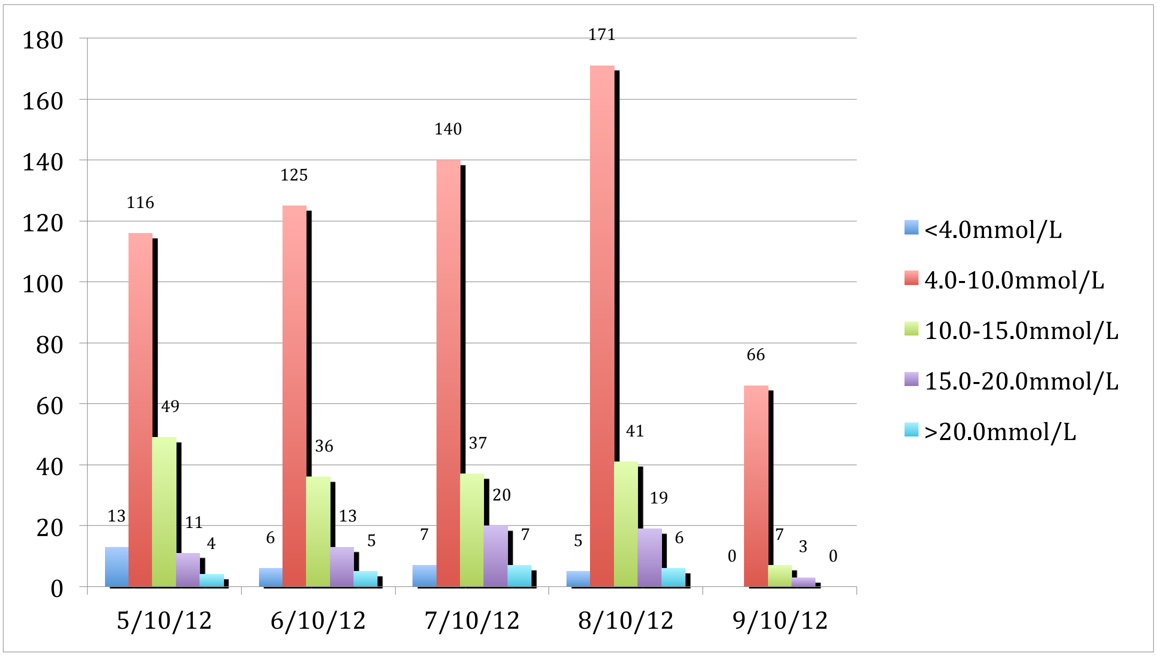

Figure S3 showing glucose recordings stratified by date and glucose level.

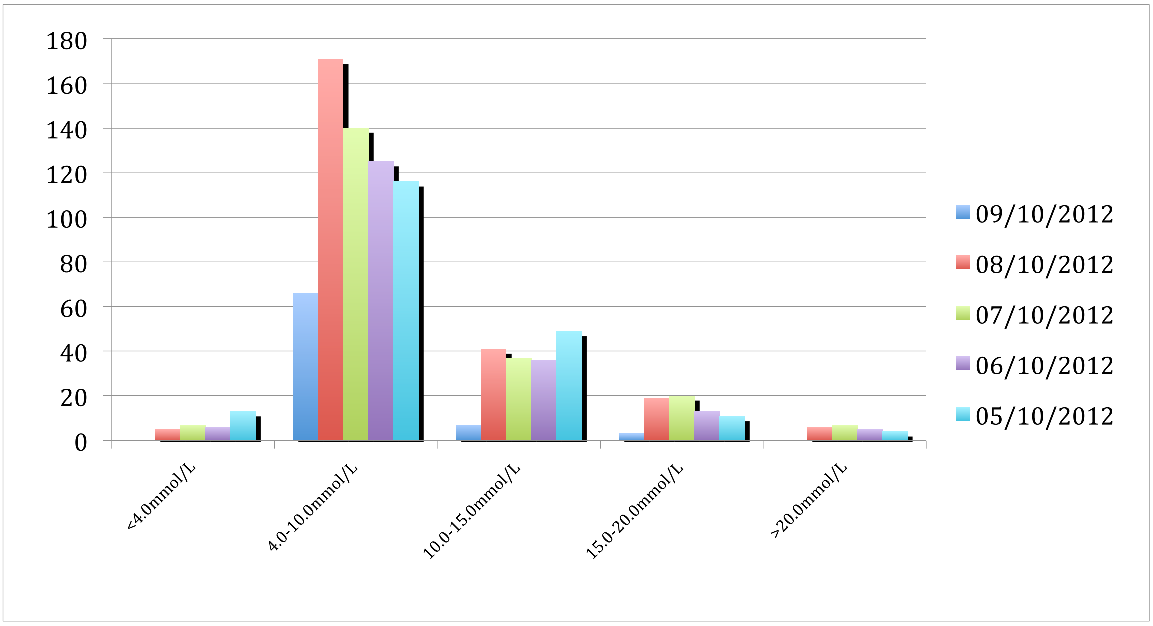

Table S2 and Figure S4 show the number of individual patients who were either moderately or severely hyperglycaemic at any point in time during the course of a day. The highest reading was taken per patient each day. The most cases of moderate-severe hyperglycaemia occurred on Sunday and Monday.

Table S2 showing number of patients with moderate or severe hyperglycaemia per day.

Glucose Level [mmol/L]	Total	09/10/2012	08/10/2012	07/10/2012	06/10/2012	05/10/2012
15.0-20.0	39	3	10	10	7	9
>20.0	13	0	4	3	3	3

Figure S4 showing number of patients with moderate or severe hyperglycaemia per day.

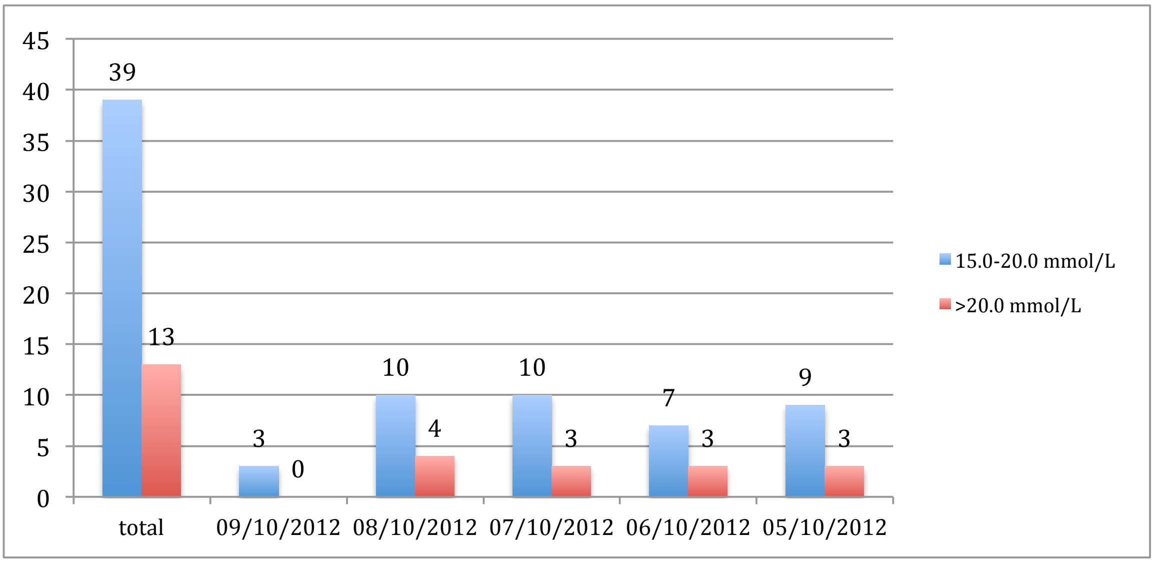

The majority of glucose readings in the moderate-severe range occurred on 4 wards [J, L, R and T]. R and T had the most cases of severe hyperglycaemia [see figure S5].

Figure S5 showing number of recordings within moderate or severe hyperglycaemia levels based on ward location.

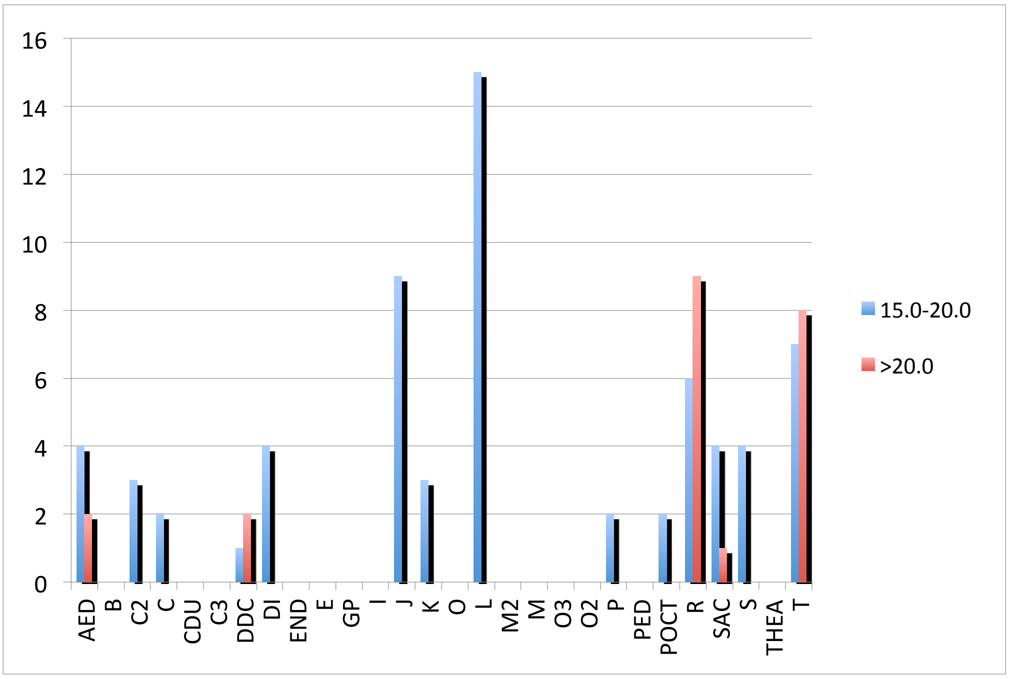

When glucose readings were analyzed according to individual patients who had either moderate or severe hyperglycaemia, average time to subsequent glucose recording was up to 6 hours and average time to normalization or glucose was up to 10 hours. This is contrary to most targets in the standards of care for the management of inpatient hyperglycaemia. One case took up to 3 days for glucose levels to fall below 15 mmol/L. A few cases were well-managed with the minimum time to glucose normalization being 49 minutes.

Table S3 showing summary statistics for time to next glucometer reading and time to normalization of glucose levels for all patients with moderate or severe hyperglycaemia.

	Time to next glucometer reading	Time to glucose <15 mmol/L
Max (hh:mm:ss)	32:52:00	75:58:00
Min (hh:mm:ss)	0:02:00	0:49:00
Mean (hh:mm:ss)	5:50:19	9:53:15
Standard Deviation (hh:mm:ss)	5:54:47	12:58:52
Variance (hh:mm:ss)	1:27:25	7:01:17

Figures S6 through S10 highlight individual cases of hypo- and hyperglycaemia as they occurred over the 5 day audit window. They show the issues of widely fluctuating glucose values and the problem of hypoglycaemia when trying to achieve tight glycaemic control. They clearly indicate a need for better protocols and an integrated care pathway for the management of inpatients with hyperglycaemia.

Figure S6: Patient with moderate hyperglycaemia. Management of this patient was complicated by hypoglycaemia but adequate control was achieved within 36 hours.

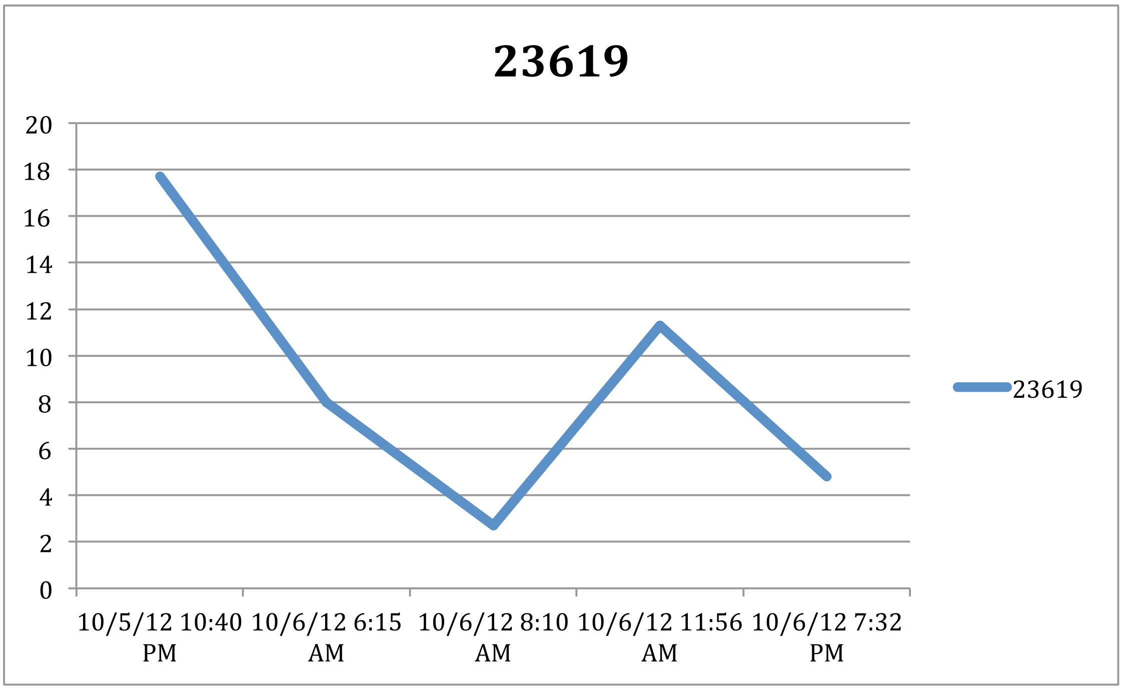

Figure S7: Case showing two instances of hypoglycaemia which resolved [either spontaneously or with appropriate management] within 1-3 hours.

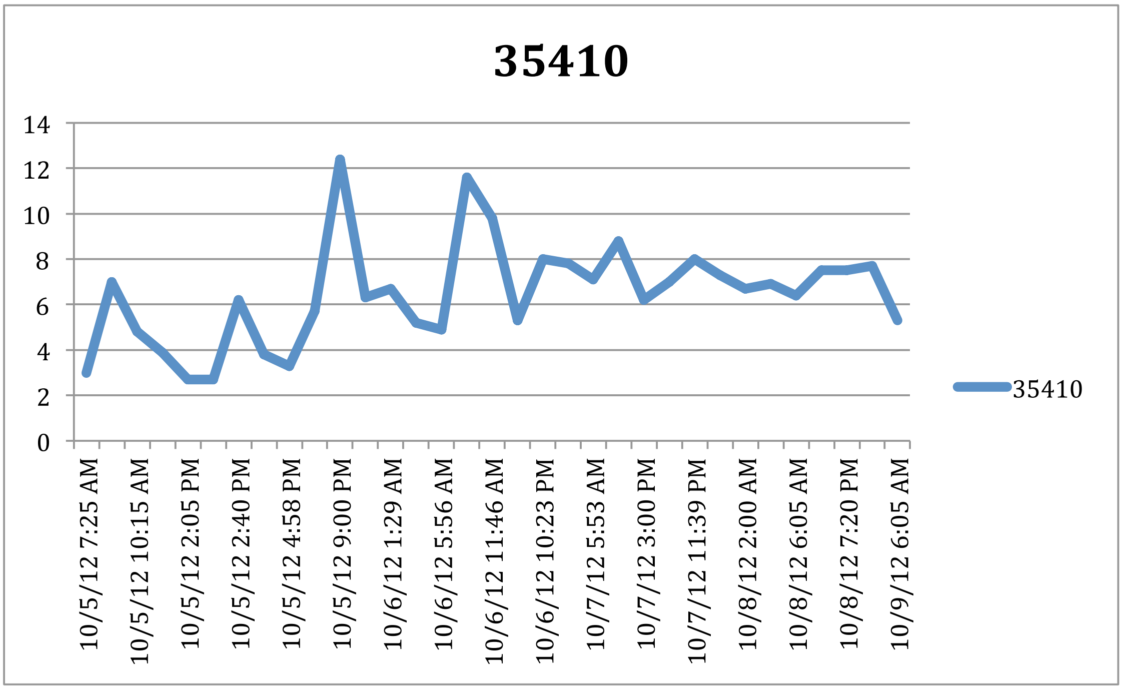

Figure S8: Inpatient with poor glycaemic control over the course of 5 days.

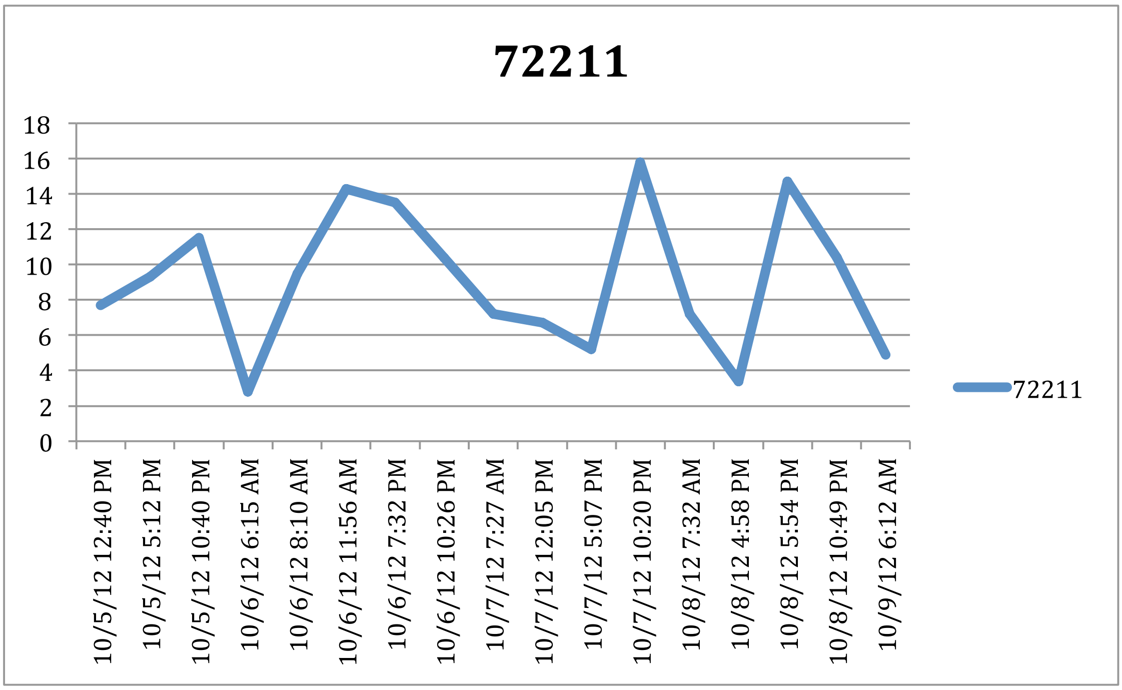

Figure S9: Case of severe hyperglycaemia and moderate hyperglycaemia in the same inpatient during a 5 day window.

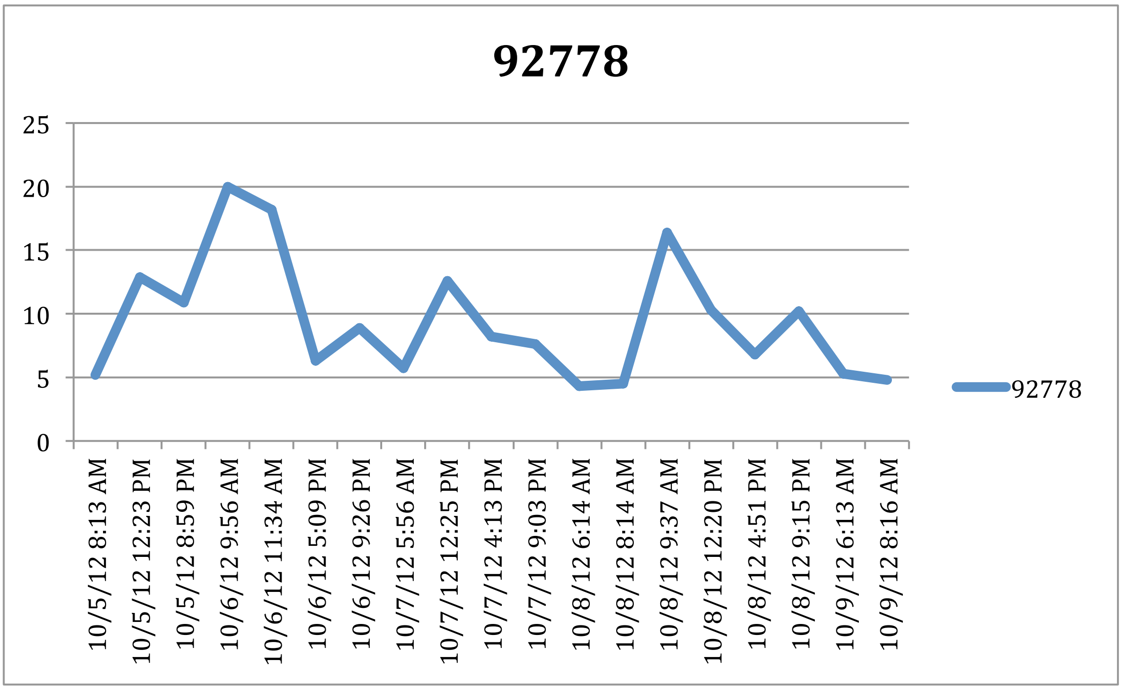

Figure S10: Inpatient with wide fluctuations in glucose levels indicating poor management regimen.

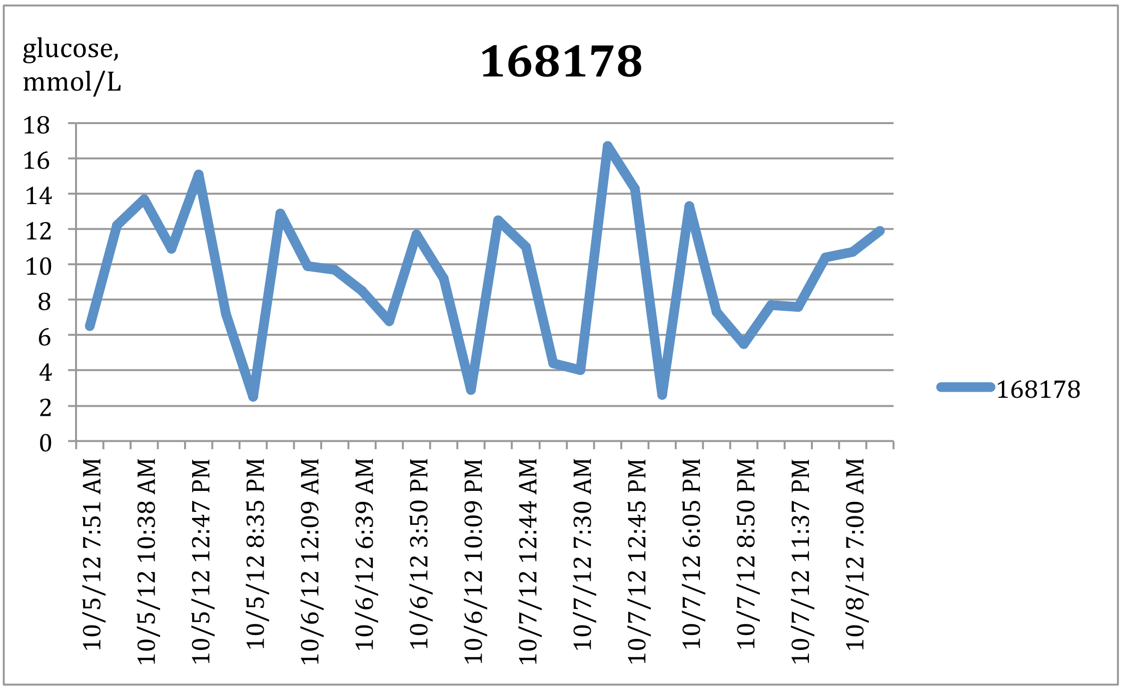

Conclusions

A large proportion [30%] of glucose readings were above the AACE/ADA recommended cutoff target of 180mg/dl [10.0 mmol/L] for management of inpatient hyperglycaemia. 52 patients had at least moderate or severe hyperglycaemia. Three wards were idenitified with large numbers of hyperglycaemic recordings. When glucose readings in the moderate-severe hyperglycaemia range were identified, there were significant delays in subsequent glucose measurement and in achieving tight glycaemic control.

Recommendations and Action Plan

1. Daily reviews of glucose recordings by laboratory POCT manager or other nominated individual.
2. Red flag system all glucose recordings within the moderate-severe hyperglycaemia range are notified to the relevant ward team, nursing staff, Diabetic Nurse Specialist. Recommendations will be made by Diabetic Nurse Specialist and POCT team regarding the need for alerting the hospital Diabetology Team.
3. Diabetes Care Bundles will be easily accessible on all wards by use of concise flow diagrams with adequate information about treatment of inpatient hypo- and hyperglycaemia with treatment targets highlighted. This will aid standardization of patient care across all healthcare staff.

Recommended POCT Glucometry intervals will be provided based on patients' clinical history, reason for admission, clinical status and prior glucose values. Re-audit at 6-monthly intervals to evaluate effectivess of the above strategies. This should address the limitations of this audit, including lack of access to patient history. Re-audit should be done in close liaison with Diabetology Team and Diabetic Nurse Specialists.

References

1. Inzucchi SE. Clinical practice. Diagnosis of diabetes. N Engl J Med. 2012 Aug 9;367(6):542-50. Review.
2. Umpierrez GE, Hellman R, Korytkowski MT, et al. Management of Hyperglycaemia in Hospitalized Patients in Non-Critical Care Setting: An Endocrine Society Clinical Practice Guideline. Journal of Clinical Endocrinology & Metabolism, January 2012, 97 (1):16–38.
3. Abourizk NN, Vora CK, Verma PK. Inpatient diabetology. The new frontier. J Gen Intern Med. 2004 May;19(5 Pt 1):466-71.
4. Juneja R. Hyperglycaemia management in the hospital: about glucose targets and process improvements. Postgrad Med. 2008 Nov;120(4):38-50.
5. Schmeltz LR, Ferrise C. Glycemic management in the inpatient setting. Hosp Pract (Minneap). 2012 Apr;40(2):44-55.
6. Schmeltz LR, Ferrise C. Glycemic management in the inpatient setting. Hosp Pract (Minneap). 2012 Apr;40(2):44-55.

SUPPLEMENTARY APPENDIX 2

1. User Interface

A ribbon interface was created for user interaction. This interface is shown in Figure S1 of Supplementary Appendix 2. The Excel® data file was loaded using this interface and processed according to a start date and end date entered in the dropdown menus. The ribbon interface had buttons for generation of reports and charts outlined in the sections below.

2. Data File Creation

Daily downloads of glucometry results were done from the Cobas IT® 1000 database and saved in .xls format. Daily data files were appended to a larger Excel® Database containing results from January 1st, 2014. A sample showing the data file and headings is shown in Figure S2 of Supplementary Appendix 2.

3. Data classification by date and glucose level

Data were first filtered according to the date range entered in the ribbon interface. During the filtering process, all values stored on the database as "> 33.3" were assigned a value of 33.3 mmol/L and all values stored as "< .6" were converted to a value of 0.6 mmol/L to allow data categorization.

Data were then classified according to the following POCT-BG values:

< 4 mmol/L : Hypoglycaemia
4 - 10 mmol/L : Normoglycaemia
10 - 15 mmol/L : Mild hyperglycaemia
15 - 20 mmol/L : Moderate hyperglycaemia
> 20 mmol/L : Severe hyperglycaemia

These cutoffs were chosen from local standards for in hospital glycaemia control based on previous annual audits.

4. Generation of reports for dysglycaemic results

Reports were generated for POCT-BG results in the form of a pivot table, as shown in Figure S3 of the Supplementary Appendix. Results filters included date, ward location and glucose category or level. Results could also be collapsed by patient ID and ward location, giving summary statistics of mean, maximum, minimum and number of results by patient ID or ward location. Reports were also generated in the format shown in Figure S4 of Supplementary Appendix 2, which allowed each dysglycaemic result to be uploaded to the Laboratory Information System.

5. Generation of Charts

The ribbon interface allowed selection of three different graphical displays: 1) Bar charts showing the percentage of POCT-BG values within each category over date; 2) Bar charts showing the number and percentage of results within each glucose category over ward location; and 3) Line graphs showing all POCT-BG levels for individual patients within the date range analysed. See Figures S5-S8 of Supplementary Appendix 2 for sample graphs.

6. Alert system on tablet devices

Toshiba Encore® 8.0 inch touchscreen tablets [Toshiba Corporation, Tokyo, Japan] with the Windows® 8.1 [Microsoft, WA, USA] operating system and Microsoft® Office Home and Student 2013 [Microsoft, WA, USA] were used in this study. All devices were password protected and data was stored on an encrypted drive (J: directory) on the device. Daily uploads of the glucometry data file to the J: folder were done.

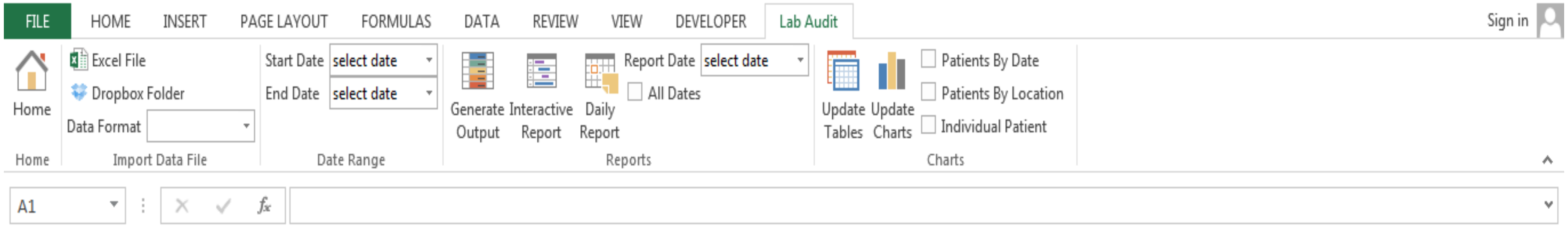

Figure S1: Ribbon User Interface for Excel® Macros

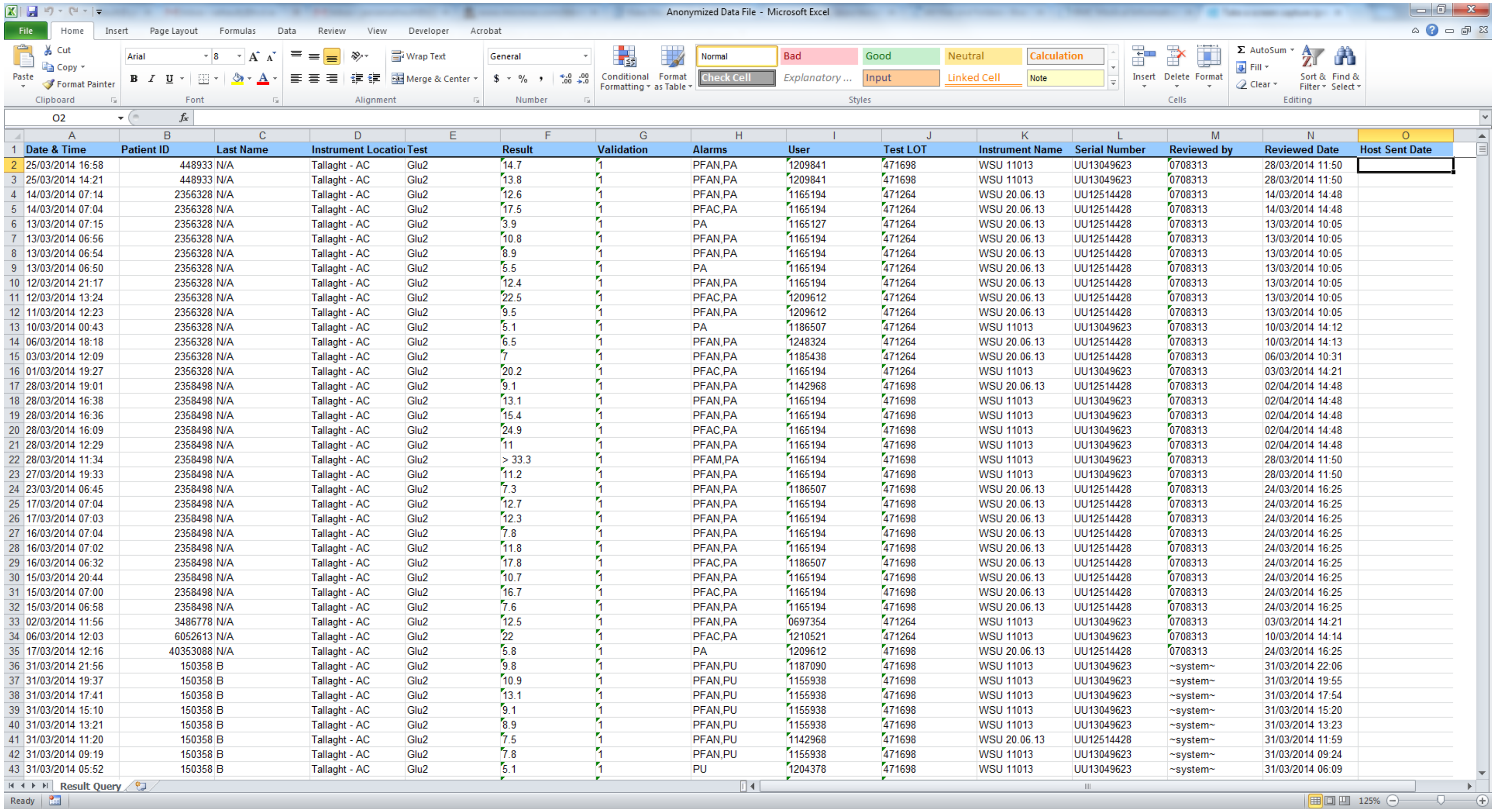

Date & Time	Patient ID	Last Name	Instrument Locatio	Test	Result	Validation	Alarms	User	Test LOT	Instrument Name	Serial Number	Reviewed by	Reviewed Date	Host Sent Date
25/03/2014 16:58	448933	N/A	Tallaght - AC	Glu2	14.7	1	PFAN,PA	1209841	471698	WSU 11013	UU13049623	0708313	28/03/2014 11:50	
25/03/2014 14:21	448933	N/A	Tallaght - AC	Glu2	13.8	1	PFAN,PA	1209841	471698	WSU 11013	UU13049623	0708313	28/03/2014 11:50	
14/03/2014 07:14	2356328	N/A	Tallaght - AC	Glu2	12.6	1	PFAN,PA	1165194	471264	WSU 20.06.13	UU12514428	0708313	14/03/2014 14:48	
14/03/2014 07:04	2356328	N/A	Tallaght - AC	Glu2	17.5	1	PFAC,PA	1165194	471264	WSU 20.06.13	UU12514428	0708313	14/03/2014 14:48	
13/03/2014 07:15	2356328	N/A	Tallaght - AC	Glu2	3.9	1	PA	1165127	471264	WSU 20.06.13	UU12514428	0708313	13/03/2014 10:05	
13/03/2014 06:56	2356328	N/A	Tallaght - AC	Glu2	10.8	1	PFAN,PA	1165194	471264	WSU 20.06.13	UU12514428	0708313	13/03/2014 10:05	
13/03/2014 06:54	2356328	N/A	Tallaght - AC	Glu2	8.9	1	PFAN,PA	1165194	471264	WSU 20.06.13	UU12514428	0708313	13/03/2014 10:05	
13/03/2014 06:50	2356328	N/A	Tallaght - AC	Glu2	5.5	1	PA	1165194	471264	WSU 20.06.13	UU12514428	0708313	13/03/2014 10:05	
12/03/2014 21:17	2356328	N/A	Tallaght - AC	Glu2	12.4	1	PFAN,PA	1165194	471264	WSU 20.06.13	UU12514428	0708313	13/03/2014 10:05	
12/03/2014 13:24	2356328	N/A	Tallaght - AC	Glu2	22.5	1	PFAC,PA	1209612	471264	WSU 20.06.13	UU12514428	0708313	13/03/2014 10:05	
11/03/2014 12:23	2356328	N/A	Tallaght - AC	Glu2	9.5	1	PFAN,PA	1209612	471264	WSU 20.06.13	UU12514428	0708313	13/03/2014 10:05	
10/03/2014 00:43	2356328	N/A	Tallaght - AC	Glu2	5.1	1	PA	1186507	471264	WSU 11013	UU13049623	0708313	10/03/2014 14:12	
06/03/2014 18:18	2356328	N/A	Tallaght - AC	Glu2	6.5	1	PFAN,PA	1248324	471264	WSU 20.06.13	UU12514428	0708313	10/03/2014 14:13	
03/03/2014 12:09	2356328	N/A	Tallaght - AC	Glu2	7	1	PFAN,PA	1185438	471264	WSU 20.06.13	UU12514428	0708313	06/03/2014 10:31	
01/03/2014 19:27	2356328	N/A	Tallaght - AC	Glu2	20.2	1	PFAC,PA	1165194	471264	WSU 11013	UU13049623	0708313	03/03/2014 14:21	
28/03/2014 19:01	2358498	N/A	Tallaght - AC	Glu2	9.1	1	PFAN,PA	1142968	471698	WSU 20.06.13	UU12514428	0708313	02/04/2014 14:48	
28/03/2014 16:38	2358498	N/A	Tallaght - AC	Glu2	13.1	1	PFAN,PA	1165194	471698	WSU 11013	UU13049623	0708313	02/04/2014 14:48	
28/03/2014 16:36	2358498	N/A	Tallaght - AC	Glu2	15.4	1	PFAN,PA	1165194	471698	WSU 11013	UU13049623	0708313	02/04/2014 14:48	
28/03/2014 16:09	2358498	N/A	Tallaght - AC	Glu2	24.9	1	PFAC,PA	1165194	471698	WSU 11013	UU13049623	0708313	02/04/2014 14:48	
28/03/2014 12:29	2358498	N/A	Tallaght - AC	Glu2	11	1	PFAN,PA	1165194	471698	WSU 11013	UU13049623	0708313	02/04/2014 14:48	
28/03/2014 11:34	2358498	N/A	Tallaght - AC	Glu2	> 33.3	1	PFAM,PA	1165194	471698	WSU 11013	UU13049623	0708313	28/03/2014 11:50	
27/03/2014 19:33	2358498	N/A	Tallaght - AC	Glu2	11.2	1	PFAN,PA	1165194	471698	WSU 11013	UU13049623	0708313	28/03/2014 11:50	
23/03/2014 06:45	2358498	N/A	Tallaght - AC	Glu2	7.3	1	PFAN,PA	1186507	471698	WSU 20.06.13	UU12514428	0708313	24/03/2014 16:25	
17/03/2014 07:04	2358498	N/A	Tallaght - AC	Glu2	12.7	1	PFAN,PA	1165194	471698	WSU 20.06.13	UU12514428	0708313	24/03/2014 16:25	
17/03/2014 07:03	2358498	N/A	Tallaght - AC	Glu2	12.3	1	PFAN,PA	1165194	471698	WSU 20.06.13	UU12514428	0708313	24/03/2014 16:25	
16/03/2014 07:04	2358498	N/A	Tallaght - AC	Glu2	7.8	1	PFAN,PA	1165194	471698	WSU 20.06.13	UU12514428	0708313	24/03/2014 16:25	
16/03/2014 07:02	2358498	N/A	Tallaght - AC	Glu2	11.8	1	PFAN,PA	1165194	471698	WSU 20.06.13	UU12514428	0708313	24/03/2014 16:25	
16/03/2014 06:32	2358498	N/A	Tallaght - AC	Glu2	17.8	1	PFAC,PA	1186507	471698	WSU 20.06.13	UU12514428	0708313	24/03/2014 16:25	
15/03/2014 20:44	2358498	N/A	Tallaght - AC	Glu2	10.7	1	PFAN,PA	1165194	471698	WSU 20.06.13	UU12514428	0708313	24/03/2014 16:25	
15/03/2014 07:00	2358498	N/A	Tallaght - AC	Glu2	16.7	1	PFAC,PA	1165194	471698	WSU 20.06.13	UU12514428	0708313	24/03/2014 16:25	
15/03/2014 06:58	2358498	N/A	Tallaght - AC	Glu2	7.6	1	PFAN,PA	1165194	471698	WSU 20.06.13	UU12514428	0708313	24/03/2014 16:25	
02/03/2014 11:56	3486778	N/A	Tallaght - AC	Glu2	12.5	1	PFAN,PA	0697354	471264	WSU 11013	UU13049623	0708313	03/03/2014 14:21	
06/03/2014 12:03	6052613	N/A	Tallaght - AC	Glu2	22	1	PFAC,PA	1210521	471264	WSU 11013	UU13049623	0708313	10/03/2014 14:14	
17/03/2014 12:16	40353088	N/A	Tallaght - AC	Glu2	5.8	1	PA	1209612	471698	WSU 20.06.13	UU12514428	0708313	24/03/2014 16:25	
31/03/2014 21:56	150358	B	Tallaght - AC	Glu2	9.8	1	PFAN,PU	1187090	471698	WSU 11013	UU13049623	~system~	31/03/2014 22:06	
31/03/2014 19:37	150358	B	Tallaght - AC	Glu2	10.9	1	PFAN,PU	1155938	471698	WSU 11013	UU13049623	~system~	31/03/2014 19:55	
31/03/2014 17:41	150358	B	Tallaght - AC	Glu2	13.1	1	PFAN,PU	1155938	471698	WSU 11013	UU13049623	~system~	31/03/2014 17:54	
31/03/2014 15:10	150358	B	Tallaght - AC	Glu2	9.1	1	PFAN,PU	1155938	471698	WSU 11013	UU13049623	~system~	31/03/2014 15:20	
31/03/2014 13:21	150358	B	Tallaght - AC	Glu2	8.9	1	PFAN,PU	1155938	471698	WSU 11013	UU13049623	~system~	31/03/2014 13:23	
31/03/2014 11:20	150358	B	Tallaght - AC	Glu2	7.5	1	PFAN,PU	1142968	471698	WSU 20.06.13	UU12514428	~system~	31/03/2014 11:59	
31/03/2014 09:19	150358	B	Tallaght - AC	Glu2	7.8	1	PFAN,PU	1155938	471698	WSU 11013	UU13049623	~system~	31/03/2014 09:24	
31/03/2014 05:52	150358	B	Tallaght - AC	Glu2	5.1	1	PU	1204378	471698	WSU 11013	UU13049623	~system~	31/03/2014 06:09	

Figure S2: Anonymised sample data file extracted from Cobas IT®

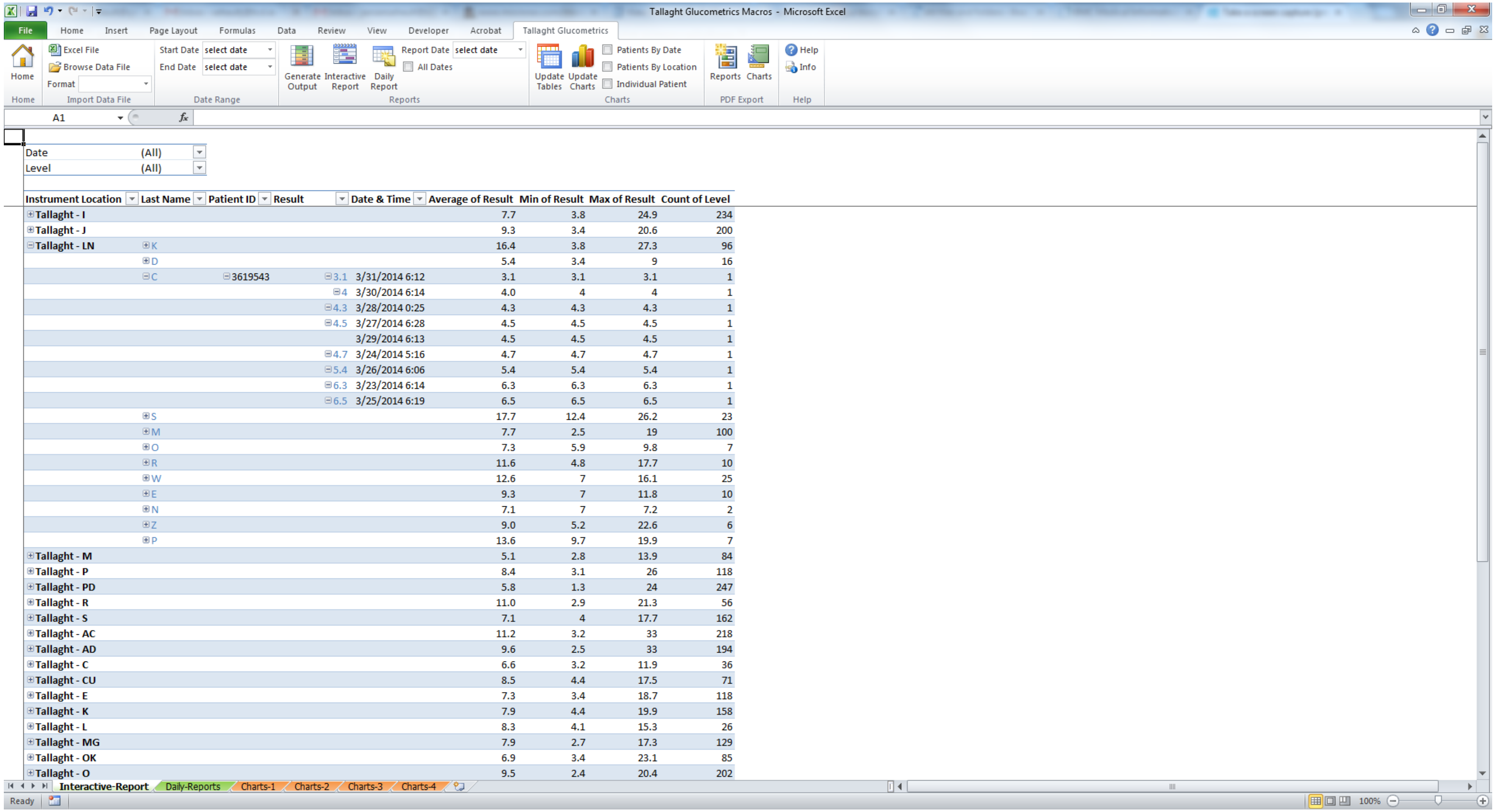

Instrument Location	Last Name	Patient ID	Result	Date & Time	Average of Result	Min of Result	Max of Result	Count of Level
Tallaght - I					7.7	3.8	24.9	234
Tallaght - J					9.3	3.4	20.6	200
Tallaght - LN	K				16.4	3.8	27.3	96
	D				5.4	3.4	9	16
	C	3619543	3.1	3/31/2014 6:12	3.1	3.1	3.1	1
			4	3/30/2014 6:14	4.0	4	4	1
			4.3	3/28/2014 0:25	4.3	4.3	4.3	1
			4.5	3/27/2014 6:28	4.5	4.5	4.5	1
				3/29/2014 6:13	4.5	4.5	4.5	1
			4.7	3/24/2014 5:16	4.7	4.7	4.7	1
			5.4	3/26/2014 6:06	5.4	5.4	5.4	1
			6.3	3/23/2014 6:14	6.3	6.3	6.3	1
			6.5	3/25/2014 6:19	6.5	6.5	6.5	1
	S				17.7	12.4	26.2	23
	M				7.7	2.5	19	100
	O				7.3	5.9	9.8	7
	R				11.6	4.8	17.7	10
	W				12.6	7	16.1	25
	E				9.3	7	11.8	10
	N				7.1	7	7.2	2
	Z				9.0	5.2	22.6	6
	P				13.6	9.7	19.9	7
Tallaght - M					5.1	2.8	13.9	84
Tallaght - P					8.4	3.1	26	118
Tallaght - PD					5.8	1.3	24	247
Tallaght - R					11.0	2.9	21.3	56
Tallaght - S					7.1	4	17.7	162
Tallaght - AC					11.2	3.2	33	218
Tallaght - AD					9.6	2.5	33	194
Tallaght - C					6.6	3.2	11.9	36
Tallaght - CU					8.5	4.4	17.5	71
Tallaght - E					7.3	3.4	18.7	118
Tallaght - K					7.9	4.4	19.9	158
Tallaght - L					8.3	4.1	15.3	26
Tallaght - MG					7.9	2.7	17.3	129
Tallaght - OK					6.9	3.4	23.1	85
Tallaght - O					9.5	2.4	20.4	202

Figure S3: Anonymised interactive report generated by Macros. Adjustable data fields include “Date”, “Glucose Level”, “Instrument Location” and “Last Name/ Patient ID”.

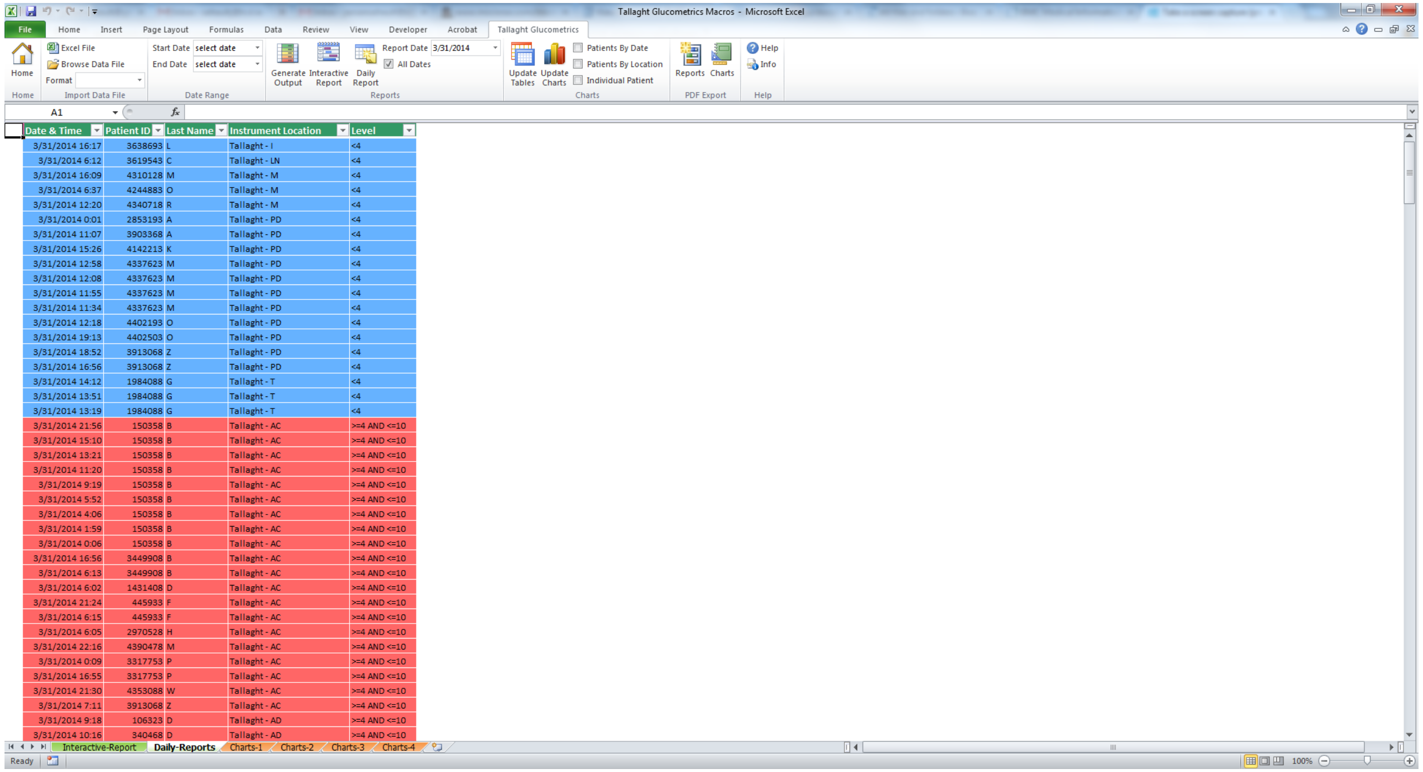

Date & Time	Patient ID	Last Name	Instrument Location	Level
3/31/2014 16:17	3638693	L	Tallaght - I	<4
3/31/2014 6:12	3619543	C	Tallaght - LN	<4
3/31/2014 16:09	4310128	M	Tallaght - M	<4
3/31/2014 6:37	4244883	O	Tallaght - M	<4
3/31/2014 12:20	4340718	R	Tallaght - M	<4
3/31/2014 0:01	2853193	A	Tallaght - PD	<4
3/31/2014 11:07	3903368	A	Tallaght - PD	<4
3/31/2014 15:26	4142213	K	Tallaght - PD	<4
3/31/2014 12:58	4337623	M	Tallaght - PD	<4
3/31/2014 12:08	4337623	M	Tallaght - PD	<4
3/31/2014 11:55	4337623	M	Tallaght - PD	<4
3/31/2014 11:34	4337623	M	Tallaght - PD	<4
3/31/2014 12:18	4402193	O	Tallaght - PD	<4
3/31/2014 19:13	4402503	O	Tallaght - PD	<4
3/31/2014 18:52	3913068	Z	Tallaght - PD	<4
3/31/2014 16:56	3913068	Z	Tallaght - PD	<4
3/31/2014 14:12	1984088	G	Tallaght - T	<4
3/31/2014 13:51	1984088	G	Tallaght - T	<4
3/31/2014 13:19	1984088	G	Tallaght - T	<4
3/31/2014 21:56	150358	B	Tallaght - AC	>=4 AND <=10
3/31/2014 15:10	150358	B	Tallaght - AC	>=4 AND <=10
3/31/2014 13:21	150358	B	Tallaght - AC	>=4 AND <=10
3/31/2014 11:20	150358	B	Tallaght - AC	>=4 AND <=10
3/31/2014 9:19	150358	B	Tallaght - AC	>=4 AND <=10
3/31/2014 5:52	150358	B	Tallaght - AC	>=4 AND <=10
3/31/2014 4:06	150358	B	Tallaght - AC	>=4 AND <=10
3/31/2014 1:59	150358	B	Tallaght - AC	>=4 AND <=10
3/31/2014 0:06	150358	B	Tallaght - AC	>=4 AND <=10
3/31/2014 16:56	3449908	B	Tallaght - AC	>=4 AND <=10
3/31/2014 6:13	3449908	B	Tallaght - AC	>=4 AND <=10
3/31/2014 6:02	1431408	D	Tallaght - AC	>=4 AND <=10
3/31/2014 21:24	445933	F	Tallaght - AC	>=4 AND <=10
3/31/2014 6:15	445933	F	Tallaght - AC	>=4 AND <=10
3/31/2014 6:05	2970528	H	Tallaght - AC	>=4 AND <=10
3/31/2014 22:16	4390478	M	Tallaght - AC	>=4 AND <=10
3/31/2014 0:09	3317753	P	Tallaght - AC	>=4 AND <=10
3/31/2014 16:55	3317753	P	Tallaght - AC	>=4 AND <=10
3/31/2014 21:30	4353088	W	Tallaght - AC	>=4 AND <=10
3/31/2014 7:11	3913068	Z	Tallaght - AC	>=4 AND <=10
3/31/2014 9:18	106323	D	Tallaght - AD	>=4 AND <=10
3/31/2014 10:16	340468	D	Tallaght - AD	>=4 AND <=10

Figure S4: Anonymised sample report by day for uploading to the LIS.

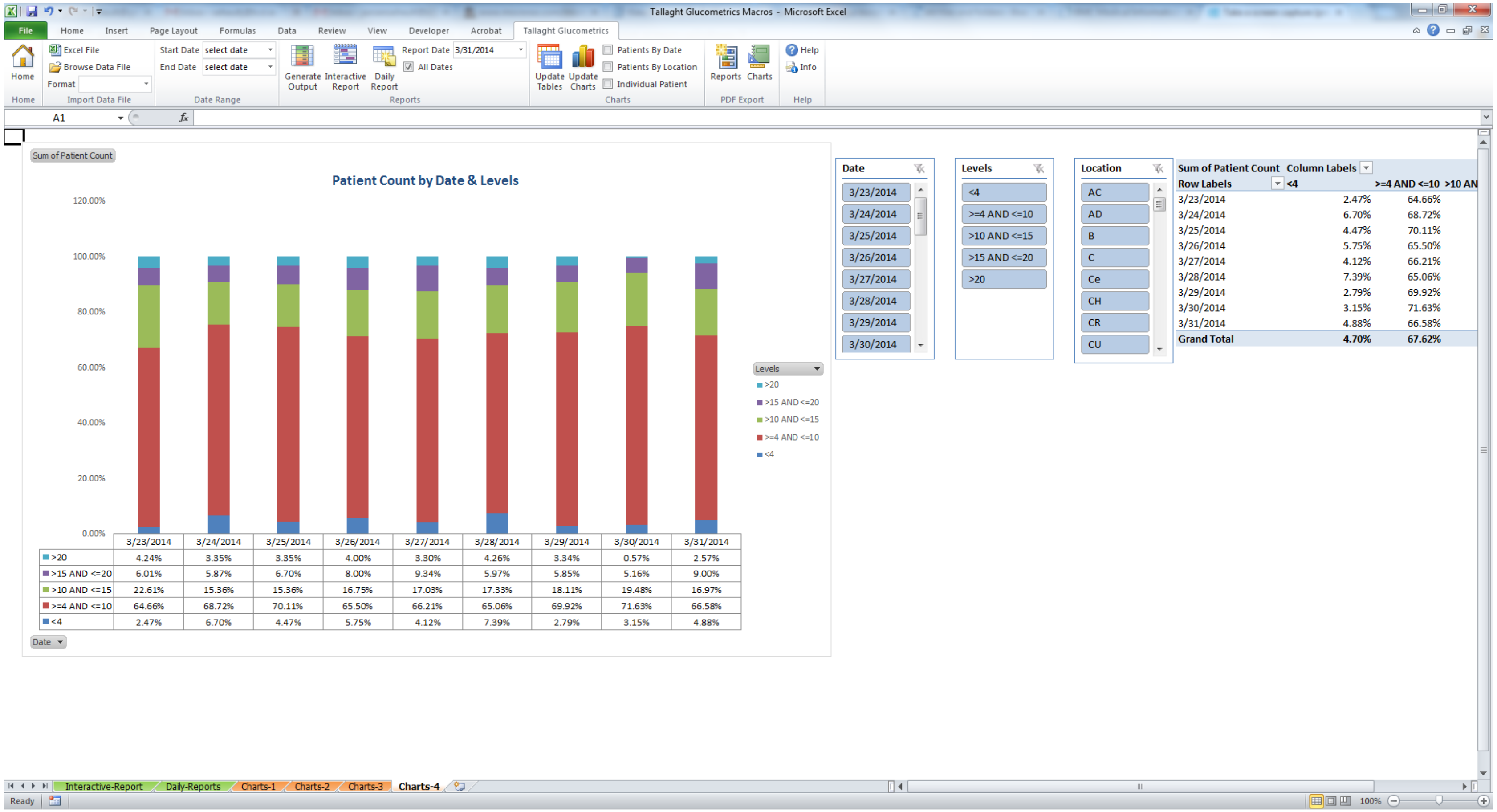

Figure S5: Vertical bar graph showing the percentage of results in each glucose level or category by date. Adjustable fields include "Instrument Location", "Glucose Level" and "Date"

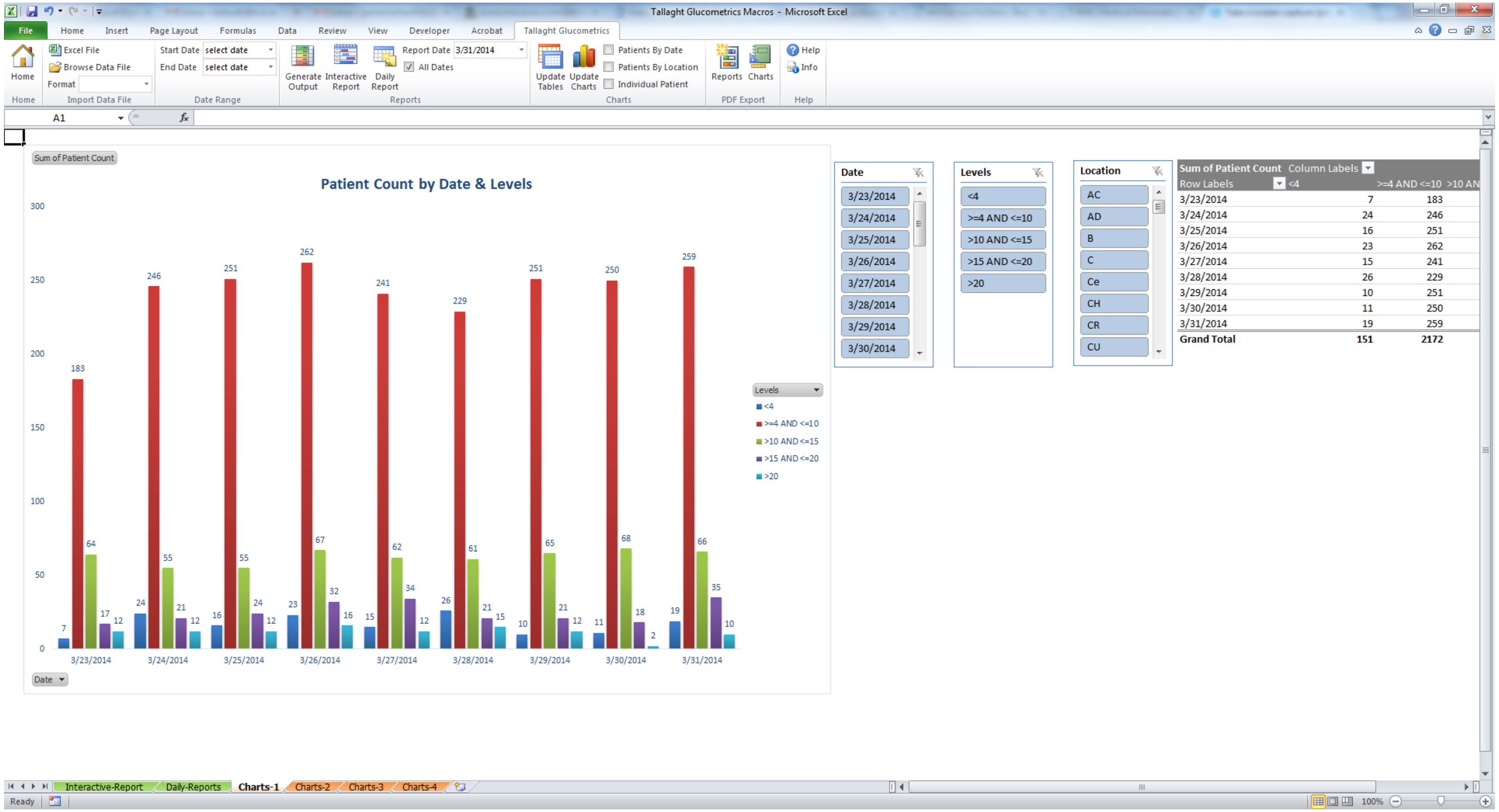

Figure S6: Vertical bar graph showing the number of results in each glucose level or category by date. Adjustable fields include “Instrument Location”, “Glucose Level” and “Date”

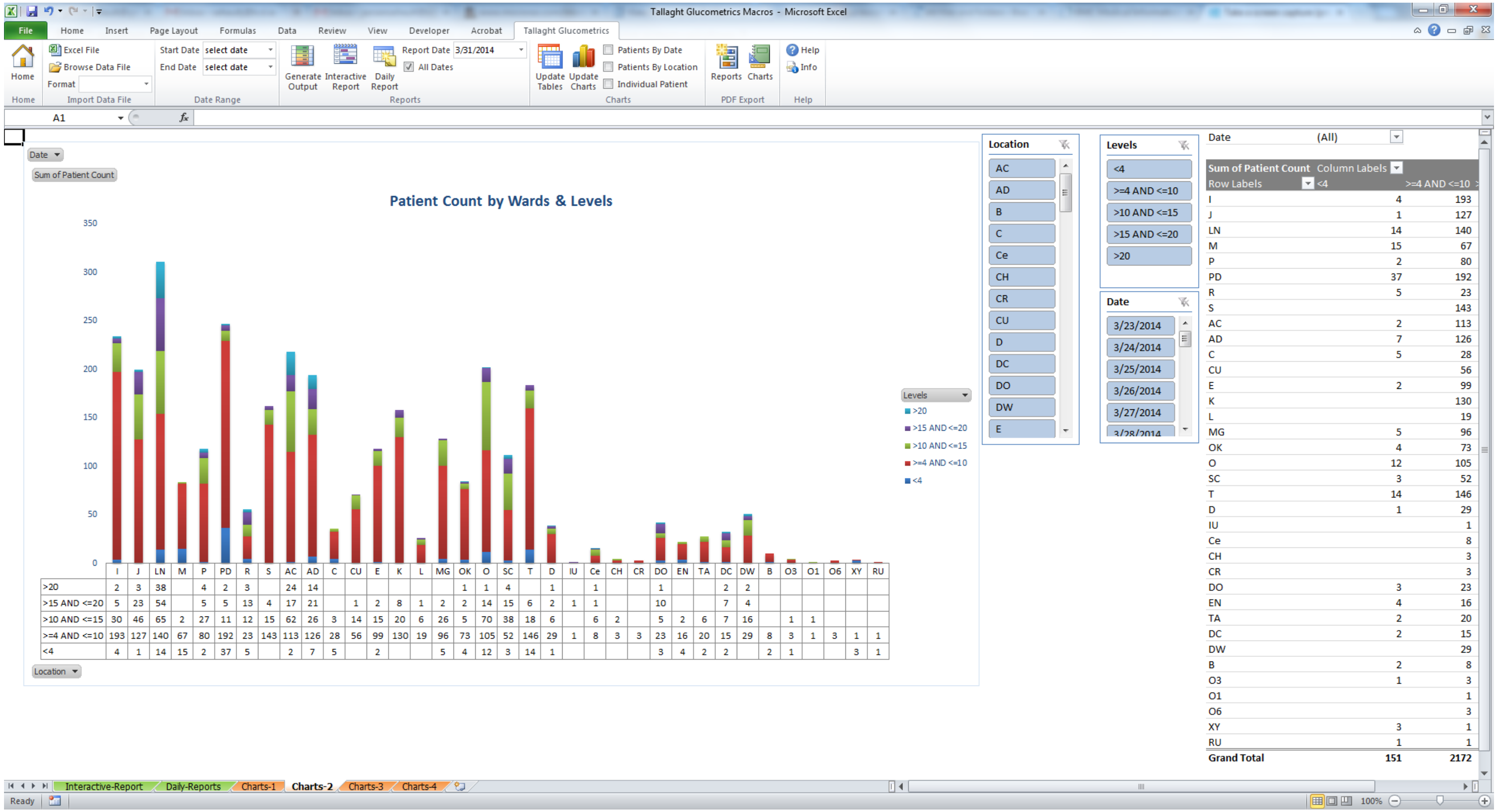

Figure S7: Stacked vertical bar graph showing the number of results in each glucose level by Ward location. Adjustable fields include “Instrument Location”, “Glucose Level” and “Date”.

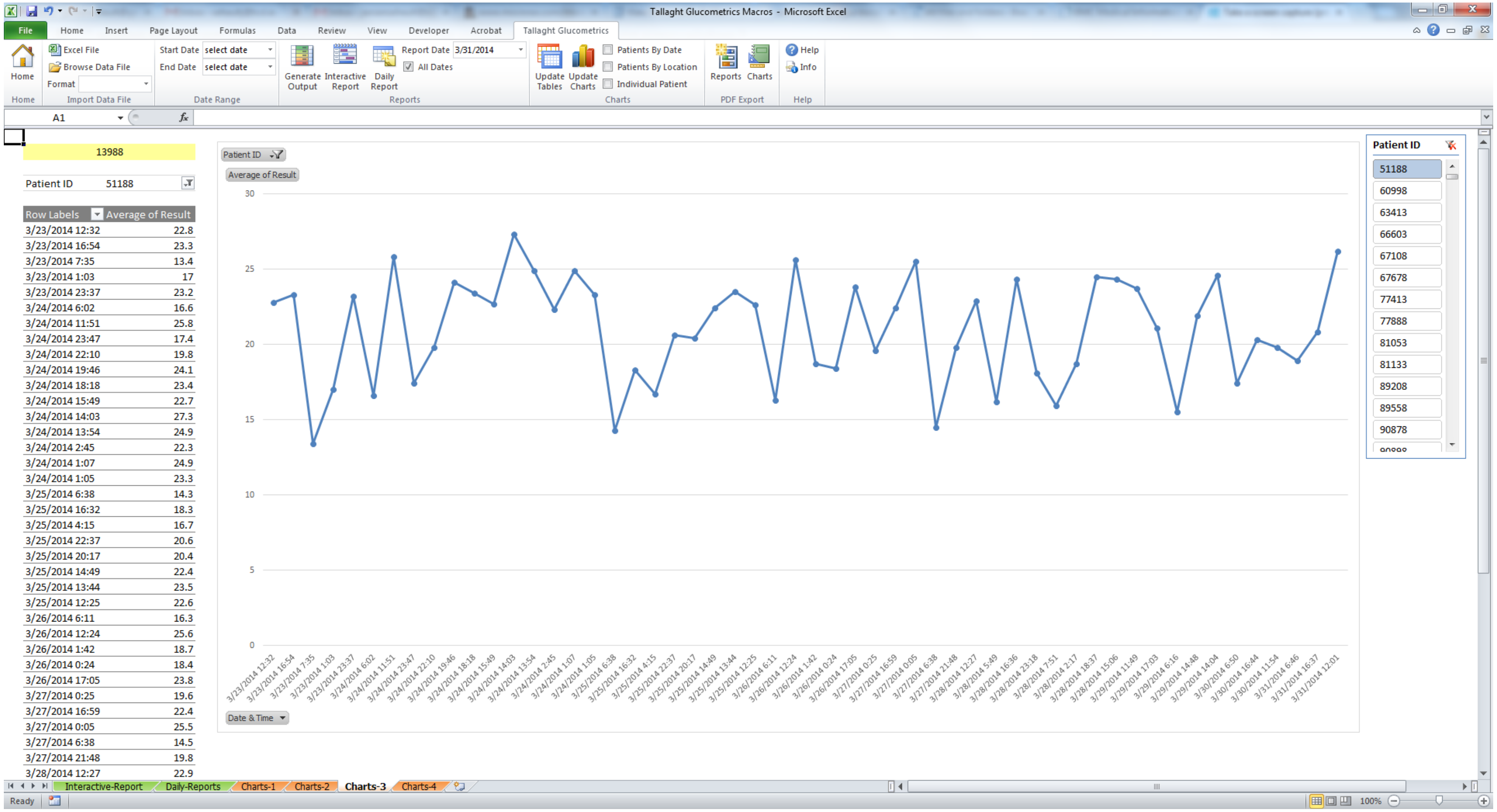

Figure S8: Line graph showing all POCT-BG results for a single patient over a specified date range. Results are listed in tabular form on the left of the graph. Patients can be selected by typing the patient ID into the yellow box or from the scrollable list (anonymised) on the right.
